# Oral Health, Inflammation, and the Burden of Multiple Long-Term Conditions: Cross-Sectional Analyses from UK Biobank and NHANES

**DOI:** 10.3390/jcm15114029

**Published:** 2026-05-22

**Authors:** Nisachon Siripaiboonpong, Jeanie Suvan, Praveen Sharma, Attawood Lertpimonchai, Crystal Marruganti, Francesco D’Aiuto

**Affiliations:** 1 Periodontology Unit, UCL Eastman Dental Institute, London WC1E 6BT, UK; nisachon.siripaiboonpong.22@ucl.ac.uk; 2 Department of Periodontology, Faculty of Dentistry, Chulalongkorn University, Bangkok 10120, Thailand; attawood.l@chula.ac.th; 3 Oral Sciences Research Group, Glasgow Dental School, School of Medicine, Dentistry & Nursing, College of Medical, Veterinary & Life Sciences, University of Glasgow, Glasgow G2 3JZ, UK; jeanie.suvan@glasgow.ac.uk; 4 Periodontal Research Group, Institute of Clinical Sciences, University of Birmingham and Birmingham Community Healthcare NHS Foundation Trust, Birmingham B5 7EG, UK; p.sharma@bham.ac.uk; 5 NIHR Birmingham Biomedical Research Centre in Inflammation, Birmingham B15 2TH, UK; 6 Unit of Periodontology, Endodontology, and Restorative Dentistry, Department of Medical Biotechnologies, University of Siena, 53100 Siena, Italy; marruganti@gmail.com; 7 Imperial College Healthcare NHS Trust, London W6 8RF, UK

**Keywords:** periodontitis, multiple long-term conditions, inflammatory mediators, NHANES, Biobank

## Abstract

**Background:** The contribution of oral inflammatory conditions to systemic disease burden remains underexplored within multimorbidity frameworks. Emerging evidence suggests that periodontal inflammation may play a role in the clustering of chronic diseases, yet few studies have evaluated this at a population level using robust datasets. The aims of this study were to investigate whether periodontal diseases are associated with Multiple long-term conditions (MLTCs) burden and severity in two population-based cohorts and to examine whether systemic inflammatory biomarkers mediate these associations. **Materials and Methods:** We analyzed two population-based cohorts: the UK Biobank (UKB; n = 500,612) and the US National Health and Nutrition Examination Survey (NHANES; n = 10,714). MLTCs were defined as the coexistence of ≥2 chronic diseases. Associations between periodontal diseases and MLTCs were assessed using multivariable logistic and multinomial logistic regression. Causal mediation analyses examined the contribution of systemic inflammatory markers. **Results:** Approximately half of all participants had MLTCs. The prevalence of periodontal diseases was 17.8% in UKB (self-reported symptoms), and 42.3% in NHANES (clinically assessed). Periodontal diseases were independently associated with greater odds of MLTCs in both UKB (OR 1.12; 95% CI 1.10–1.14) and NHANES (OR 1.22; 95% CI 1.09–1.37). Associations were stronger among adults aged ≤ 60 years. A consistent dose-response relationship was observed between periodontal status and the number and severity of chronic conditions, as well as inflammatory-related MLTCs. Mediation analyses suggested partial effects through white blood cell count, neutrophils, and C-reactive protein. **Conclusions:** Periodontal inflammation is independently associated with greater multimorbidity burden, particularly in younger adults. Systemic inflammation may offer a plausible biological link, and these findings position oral health as an underrecognized and modifiable target in multimorbidity prevention and management frameworks, warranting prospective investigation.

## 1. Introduction

Multiple long-term conditions (MLTCs), also commonly referred to as multimorbidity, are defined as the coexistence of two or more chronic diseases and represent a major and growing public health challenge globally. Their burden is driven by population ageing, unhealthy lifestyle factors, and widening health inequalities [1]. Globally, the prevalence of MLTCs is estimated at 37.2%, rising to over 50% among adults aged 60 years and older [2]. The burden of MLTCs has increased steadily over the past two decades, exerting profound impacts on mortality, functional status, quality of life, and healthcare costs [3]. A recent systematic review reported that individuals with two or more chronic conditions have a 1.73-fold higher odds of mortality, increasing to 2.72 times with three or more conditions [4]. These trends highlight the urgent need for health systems to shift from single-disease approaches to coordinated, patient-centered models of care.

Chronic inflammatory conditions are increasingly recognized as shared contributors to the development and progression of multiple chronic diseases. Among these, periodontal diseases—chronic inflammatory conditions affecting the tooth-supporting tissues—have garnered attention for their potential systemic relevance. Periodontitis and gingivitis affect over 60% of adults globally, with 23.6% experiencing severe disease, and their burden has nearly doubled between 1990 and 2019 [5]. Beyond oral symptoms, periodontal diseases are associated with tooth loss, impaired mastication, nutritional deficits, and reduced quality of life, as well as substantial economic costs related to treatment and productivity loss [6].

An expanding body of evidence links periodontal inflammation to systemic conditions such as diabetes, cardiovascular disease, and hypertension [7]. Proposed biological pathways include chronic systemic inflammation, direct dissemination of oral pathogens and their byproducts into the bloodstream and immune dysregulation [8]. Despite these plausible mechanisms, oral health remains largely absent from public health and clinical frameworks addressing multimorbidity. The potential association between periodontal diseases and the clustering, severity, or burden of MLTCs is not yet fully understood. Most available evidence comes from single-disease models, small or non-representative cohorts, or studies without mechanistic insights [9,10]. Clarifying the role of periodontal diseases in multimorbidity is increasingly important given the concurrent rise of MLTCs and chronic oral conditions. If confirmed, such links could open new opportunities for integrated prevention and early intervention strategies that span both medical and dental care.

In this study, we analyzed two large population-based datasets—the US National Health and Nutrition Examination Survey (NHANES) and the UK Biobank (UKB)—to investigate whether periodontal diseases are associated with MLTCs burden and severity. We further examined whether systemic inflammatory biomarkers mediate these associations. These findings aim to inform public health priorities and support the inclusion of oral health in chronic disease prevention and management strategies.

## 2. Materials and Methods

### 2.1. Study Design and Data Sources

This cross-sectional analysis draws on data from the UKB and NHANES (2009–2014) surveys. The study was designed and reported in accordance with the Strengthening the reporting of observational studies in epidemiology (STROBE) guidelines [11].

UKB recruited over 500,000 participants aged 40–69 years across the United Kingdom between 2006 and 2010, using population-based convenience sampling [12]. NHANES is a cross-sectional, nationally representative survey of the non-institutionalized U.S. population, conducted by the US Centers for Disease Control and Prevention (CDC) using a complex, multistage probability sampling design (full study protocols are available elsewhere [13]). For UKB, all participants with available data on periodontal status and chronic conditions were eligible for inclusion; no additional study-specific exclusion criteria were applied. For NHANES, participants were additionally required to be dentate adults aged 30 years or older, in accordance with the NHANES periodontal examination protocol, which excluded edentulous individuals [14]. Missing data for all other variables were low, except for household income and physical activity in UKB (Supplementary Materials: Table S4), and were handled using multiple imputation by chained equations (MICE) for UKB and complete-case analysis for NHANES, as survey-weighted analyses do not support multiple imputation.

#### 2.1.1. Exposure: Periodontal Diseases Assessment

Oral inflammatory status, as a proxy for periodontal diseases, was assessed using different methods across the two datasets. In UKB, self-reported responses to questions on bleeding gums, painful gums, and loose teeth were used to define periodontal inflammatory symptoms. A positive response to any of the items was taken to indicate current or past evidence of self-reported periodontal inflammatory symptoms [15].

In NHANES, trained and calibrated examiners conducted a whole-mouth periodontal examination among dentate adults aged 30 years or older [13]. Clinical measurements included probing gingival pocket depth (PPD) and clinical attachment level (CAL). Periodontitis was classified using standardized criteria based on the American Academy of Periodontology/CDC case definitions [16].

#### 2.1.2. Outcomes: Assessment of Multiple Long-Term Conditions

The primary outcome was the prevalence of MLTCs, defined as the coexistence of two or more chronic conditions in an individual [1]. As there is no universally accepted list of conditions used to define MLTCs, we followed established practices within each dataset [17]. In the UKB, MLTCs were identified using 41 chronic disease categories [18] through self-reported diagnoses via standardized touchscreen questionnaires and linked medical records, in accordance with National Institute for Health and Care Excellence (NICE) recommendations [19]. In NHANES, MLTCs were derived from 22 condition categories selected to align with the same NICE recommendations, based on data availability within the NHANES dataset [19]; conditions were primarily ascertained through self-report questionnaires, with clinical or laboratory assessment additionally used for a subset of conditions (full definitions in Supplementary Materials: Tables S1 and S2).

Secondary outcomes included the total number of chronic conditions per participant (categorized as 0–1, 2, 3, and ≥4 conditions), MLTCs severity, and the presence of inflammatory-related MLTCs. MLTCs severity was assessed using the Cambridge Multimorbidity Score (CMS) [20], a validated UK-derived index that assigns weights to individual conditions based on their association with primary care use and mortality risk. For conditions without an officially validated weight, severity scores were imputed based on a previous published study [18]. This continuous score supports risk stratification, and although developed in a UK setting, its use is increasingly generalizable across health systems. MLTCs severity was categorized into quartiles based on the distribution of CMS scores, as no standardized thresholds have been established (weightings are provided in the Supplementary Materials: Table S3).

As a secondary exploratory outcome, inflammatory-related MLTCs was defined as the coexistence of two or more chronic conditions characterized by chronic systemic inflammation in their pathogenesis, including asthma, chronic kidney disease, connective tissue disorders, inflammatory bowel disease, diabetes, psoriasis or eczema, cardiovascular diseases, and depression. This outcome was developed a priori based on established evidence linking systemic inflammation to the pathogenesis of these conditions [21], and was designed to examine whether associations with periodontal diseases were more pronounced when restricted to conditions sharing a common inflammatory mechanism.

#### 2.1.3. Covariates

Potential confounding variables were identified a priori, based on previous evidence [22,23], as likely to influence the association between periodontal diseases and MLTCs. These included age, gender, body mass index (BMI), physical activity, marital status, ethnicity, and smoking status across both datasets. In addition, household income and the Townsend deprivation index were included for UKB, while the acculturation score and family poverty income ratio were considered for NHANES. All covariates were included as adjustment variables in multivariable regression models.

#### 2.1.4. Inflammatory Mediators

To explore potential biological pathways underlying the association between periodontal diseases and MLTCs, a panel of blood-based biomarkers was included as candidate mediators [24,25,26]. These comprised: white blood cell (WBC) count, platelet count, lymphocyte count, monocyte count, neutrophil count, C-reactive protein (CRP), alanine transaminase (ALT), and aspartate transaminase (AST). These markers were selected based on prior evidence linking systemic inflammation and liver function to both periodontitis and multimorbidity. These biomarkers are commonly used across primary care and hospital settings to assess systemic inflammatory burden and hepatic involvement.

### 2.2. Statistical Analyses

#### 2.2.1. Data Preparation

In UKB, participants with missing data on periodontal inflammatory symptoms or MLTCs were excluded from the analysis. For other variables, including covariates and inflammatory mediators, missing values were assumed to be missing at random and addressed using MICE. Between 20 and 50 imputations were performed to account for uncertainty in the imputed values and maximize statistical efficiency [27]. For NHANES, complete-case analysis was used, as multiple imputation is not supported within complex survey-weighted analyses. Missing data across variables was low and is reported in the Supplementary Materials: Table S4.

#### 2.2.2. Descriptive Statistics

In UKB, complete-case analyses were prioritized; where missing data was present (Supplementary Materials: Table S4), imputed estimates were reported as means (standard deviation, SD) for continuous variables and number (%) for categorical variables. For NHANES, all analyses incorporated complex survey weights to account for stratification and clustering; continuous variables were summarized as means (standard error, SE) and categorical variables as weighted proportions (SE). Group comparisons between participants with and without periodontal diseases were conducted using Pearson’s chi-square tests for categorical variables, *t*-tests for continuous variables (UKB), and survey-weighted linear regression models (NHANES). Given the large sample size in UKB, robust parametric comparisons were adopted as appropriate.

#### 2.2.3. Regression Modelling

Univariate logistic regression was used for binary outcomes (MLTCs and inflammatory-related MLTCs), and univariate multinomial logistic regression for ordered outcomes (number of conditions and severity of MLTCs). Covariates were identified a priori based on previous evidence and included in all multivariable models. Adjusted odds ratios (ORs) and 95% confidence intervals (CIs) were estimated from multivariable models. Variance Inflation Factors (VIF) were calculated to assess multicollinearity among covariates, with values above 5 considered indicative of potential concern. Sensitivity analyses were conducted by stratifying participants into younger (≤60 years) and older (>60 years) age groups [28]. Subgroup analyses explored alternative case definitions for periodontal diseases: in UKB, separate models were fitted for individual symptoms (bleeding gums, painful gums, and loose teeth); in NHANES, models were additionally stratified by periodontitis severity (mild, moderate, severe) [15] and by extent of disease involvement.

#### 2.2.4. Mediation Analysis

Mediation models were constructed to examine whether inflammatory biomarkers partially mediated the association between periodontal diseases and MLTCs. The total association was decomposed into direct effects of periodontal diseases on MLTCs outcomes and indirect effects operating through each inflammatory mediator. Due to skewed distributions, all mediator variables were log-transformed prior to modelling. Mediation effects were estimated using the Paramed package in Stata [29], which implements counterfactual mediation analysis, and expressed as: the Controlled Direct Effect (CDE; direct effect with the mediator fixed at a specified level), the Natural Direct Effect (NDE; direct effect not operating through the mediator), the Natural Indirect Effect (NIE; indirect effect through the inflammatory pathway), and the Marginal Total Effect (MTE; combined direct and indirect effects) [30]. The proportion mediated was calculated as the ratio of NIE to MTE. All analyses were performed using Stata version 18.5 MP (StataCorp LLC, College Station, TX, USA) [31]. Two-sided *p*-values less than 0.05 were considered statistically significant.

## 3. Results

### 3.1. Participants’ Characteristics

A total of 500,612 UKB participants and 10,714 NHANES participants with complete data on periodontal status and chronic conditions were included in the analysis (missing data by variable reported in the Supplementary Materials).

In UKB, the mean age was 56.53 years (SD 8.09), and the proportion of females slightly exceeded that of males (54.41% vs. 45.59%). The majority of participants identified as White (94.67%), held a college degree or higher (48.83%), were never-smokers (54.75%), and lived with a partner (89.64%). The mean BMI was 27.43 kg/m^2^ (SD 4.80). Half of the participants had MLTCs (50.49%), most commonly with two conditions. The mean MLTCs severity score was 0.78 (SD 0.97). The prevalence of periodontal symptoms was 17.80%, including bleeding gums (13.25%), painful gums (3.04%) and loose teeth (4.33%) (Table 1).

In NHANES, participants were younger (mean age 50.84, SE 0.24 years), with females comprising a higher proportion (51.17%). Demographic characteristics were broadly similar to UKB, although the proportion identifying as non-Hispanic White was lower (68.39%). Most participants were non-smokers (82.59%), married (63.27%), and had a college degree or higher (63.78%). The mean BMI was higher than in UKB (29.17 ± 0.11 kg/m^2^). The prevalence of MLTCs was 50.25%, with two conditions most common. The mean MLTCs severity score was 0.55 ± 0.01. The prevalence of periodontitis was 42.26%, classified as mild (4.3%), moderate (30.1%), and severe (7.86%) (Table 2).

### 3.2. Association Between Oral Inflammation and Multiple Long-Term Conditions

**Figure 1:**
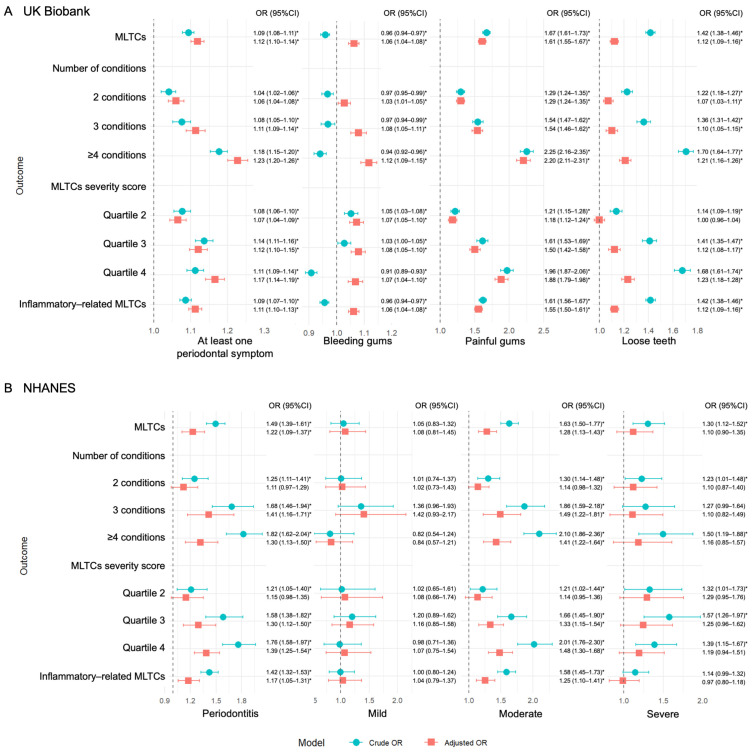
Association between periodontal conditions and multiple long-term conditions (MLTCs) outcomes. Panel (**A**) shows results from the UK Biobank (UKB) and panel (**B**) from the National Health and Nutrition Examination Survey (NHANES). Each forest plot displays crude and adjusted odds ratios (OR) with 95% confidence intervals (CI) for the association between specific periodontal exposures (columns) and MLTCs outcomes (rows). The dashed vertical line represents an OR of 1.0 (no association). * Statistically significant at *p* < 0.05.

**Figure 2:**
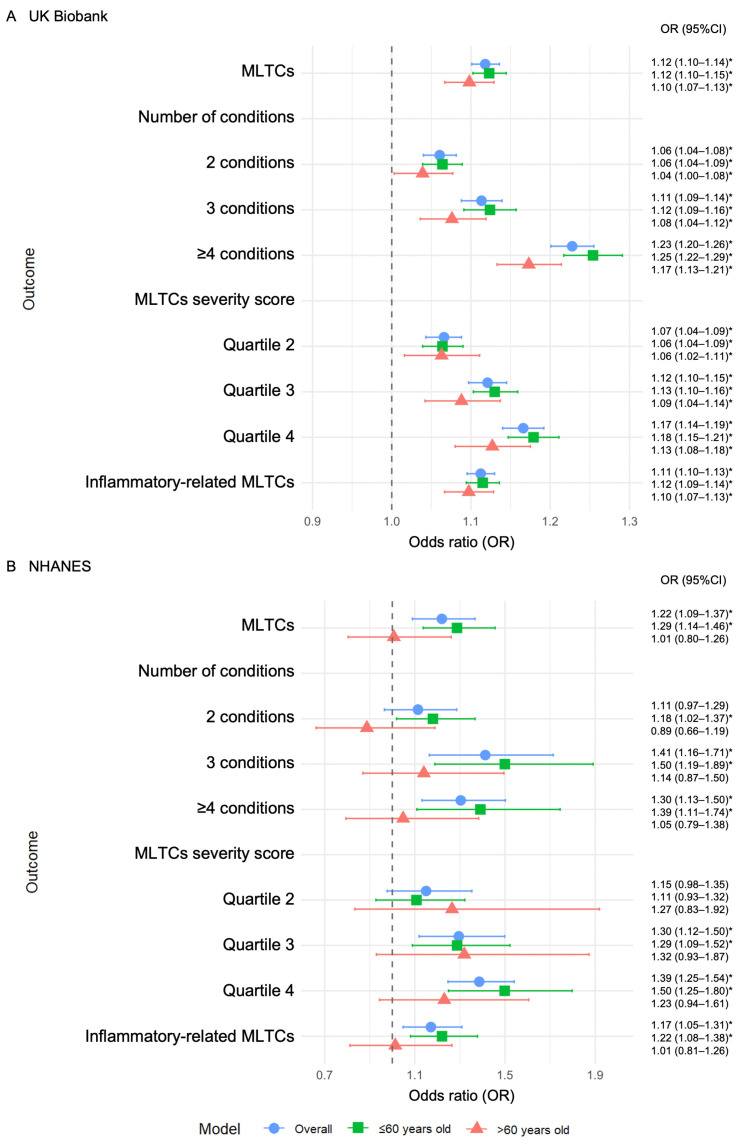
Association between periodontal conditions and multiple long-term conditions (MLTCs) stratified by age group. Panel (**A**) shows results from the UK Biobank (UKB) and panel (**B**) from the National Health and Nutrition Examination Survey (NHANES). For each outcome (rows), three adjusted odds ratios (OR) with 95% confidence intervals (CI) are displayed: overall (blue circle), ≤60 years old (green square), and >60 years old (red triangle). The dashed vertical line represents an OR of 1.0 (no association). * Statistically significant at *p* < 0.05.

In UKB, periodontal inflammatory symptoms were associated with higher odds of MLTCs (adjusted OR 1.12 [1.10–1.14], *p* < 0.001). Individually, painful gums (adjusted OR 1.61 [1.55–1.67], *p* < 0.001), bleeding gums (adjusted OR 1.06 [1.04–1.08], *p* < 0.001), and loose teeth (adjusted OR 1.12 [1.09–1.16], *p* < 0.001) were associated with MLTCs (Figure 1A). Sensitivity analyses by age group suggested a borderline stronger association among participants aged ≤60 years (adjusted OR 1.12 [1.10–1.15]) (Figure 2A).

In NHANES, similar patterns emerged (adjusted OR 1.22 [1.09–1.37], *p* = 0.001) (Figure 1B). Moderate periodontitis showed the strongest association (adjusted OR 1.28 [1.13–1.43], *p* = 0.001), while mild and severe categories did not reach statistical significance (Figure 1B). Greater extent of periodontal tissue loss (CAL ≥ 3 mm, ≥4 mm, and ≥5 mm) was associated with increased odds of MLTCs, whereas PPD-based measures were not (Supplementary Materials: Table S5). Sensitivity analysis confirmed stronger associations in younger participants (Figure 2B).

Multicollinearity diagnostics confirmed no meaningful collinearity across covariates in either dataset (mean VIF: 1.67 in UKB and 1.61 in NHANES). All variables had VIF values below 5, with the exception of the acculturation score in NHANES (VIF 5.15), which was expected given its conceptual overlap with ethnicity and was not considered to represent problematic collinearity in this context.

### 3.3. Association Between Periodontal Diseases and Number of Chronic Conditions

In UKB, periodontal inflammatory symptoms were associated with higher odds of having (a) Two conditions (adjusted OR 1.06 [1.04–1.08], *p* < 0.001), (b) Three conditions (adjusted OR 1.11 [1.09–1.14], *p* < 0.001) and (c) Four or more conditions (adjusted OR 1.23 [1.20–1.26], *p* < 0.001) (Figure 1A). A dose-response gradient was observed, with increasing number of conditions among participants reporting periodontal problems. All individual symptoms showed consistent associations. Sensitivity analyses confirmed that associations were stronger among younger participants (≤60 years) (Figure 2A).

In NHANES, periodontitis was associated with three conditions (adjusted OR 1.41 [1.16–1.71], *p* = 0.001), and four or more conditions (adjusted OR 1.30 [1.13–1.50], *p* < 0.001). Moderate periodontitis was strongly associated with higher number of conditions (Figure 1B). Greater extent of periodontal tissue loss (CAL ≥ 3 mm and ≥4 mm) remained associated with multiple conditions (Supplementary Materials: Table S5). Sensitivity analysis showed stronger effects among younger participants (Figure 2B).

### 3.4. Association Between Periodontal Diseases and Severity of Long-Term Conditions

In UKB, periodontal inflammatory symptoms were associated with increasing severity of MLTCs across CMS quartiles: Quartile 2 (adjusted OR 1.07 [1.04–1.09]), Quartile 3 (adjusted OR 1.12 [1.10–1.15]) and Quartile 4 (adjusted OR 1.17 [1.14–1.19]) (Figure 1A). Painful gums and loose teeth showed parallel dose-response patterns. Bleeding gums were also associated with severity, albeit more modestly. Sensitivity analyses confirmed stronger associations in younger individuals (Figure 2A).

In NHANES, periodontitis was associated with MLTCs severity in quartiles 3 and 4 (adjusted OR 1.30 [1.12–1.50], *p* = 0.001 and OR 1.39 [1.25–1.54], *p* < 0.001 respectively). Moderate periodontitis showed the strongest effects (Figure 1B). Greater extent of attachment loss confirmed associations across all severity quartiles, while PPD-based indicators were not predictive (Supplementary Materials: Table S5). Age-stratified analyses confirmed that associations were stronger among younger participants.

### 3.5. Association with Inflammatory-Related Long-Term Conditions

In UKB, periodontal inflammatory symptoms were associated with an 11% higher odds of inflammatory-related MLTCs. Bleeding gums (adjusted OR 1.06 [1.04–1.08]), painful gums (adjusted OR 1.55 [1.50–1.61]), and loose teeth (adjusted OR 1.12 [1.09–1.16]) were associated with inflammatory-related MLTCs (Figure 1A). In NHANES, similar associations were observed (adjusted OR 1.17 [1.05–1.31], *p* = 0.006). Moderate periodontitis showed consistent effects, as did greater CAL extent at thresholds ≥3 mm and ≥4 mm (Supplementary Materials: Table S5).

### 3.6. Mediation Analysis Results

**Figure 3:**
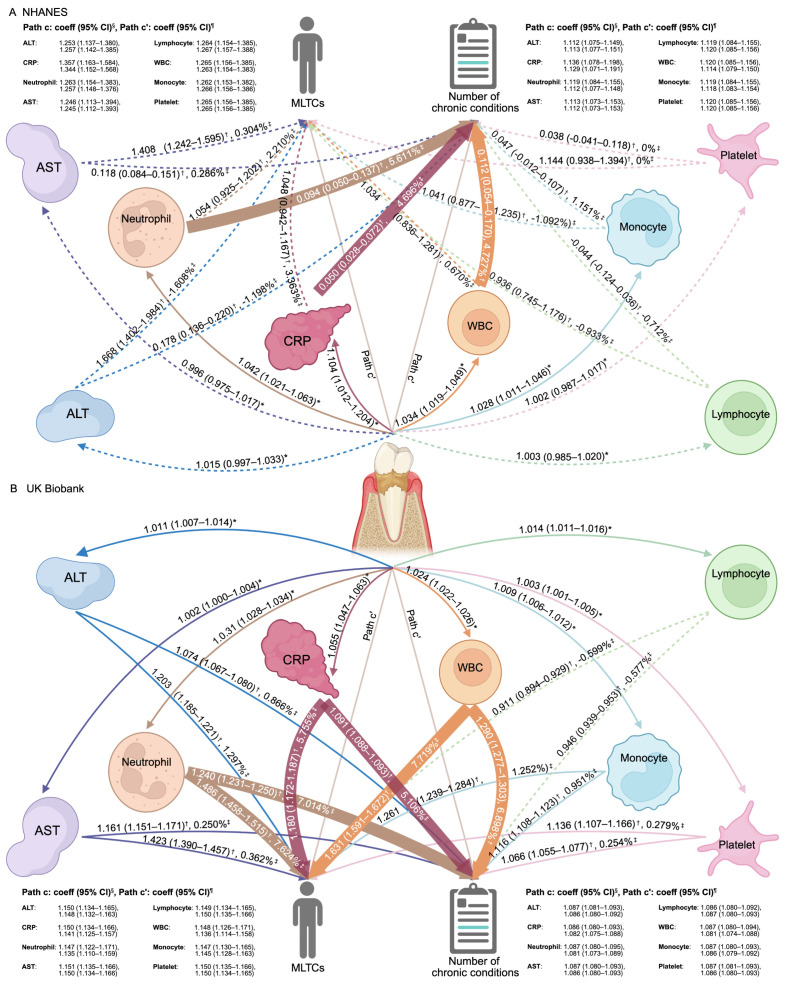
Mediation analyses results for the association between periodontal diseases and multiple long-term conditions and number of chronic conditions, shown separately for NHANES (panel (**A**)) and UK Biobank (panel (**B**)). Each diagram shows periodontal disease (center) as the exposure and MLTCs (left) or number of chronic conditions (right) as the outcomes, linked via 8 candidate inflammatory mediators (CRP, ALT, AST, WBC, neutrophils, lymphocytes, monocytes, and platelets). Four path estimates are labelled on each arrow. * Path a (exposure → mediator): association between periodontal diseases and each mediator; coefficients are exponentiated from log-transformed data. † Path b (mediator → outcome): association between each mediator and the outcome, adjusted for periodontal diseases. ‡ Proportion mediated (indirect effect): the percentage of the total effect of periodontal disease on the outcome that operates through each mediator. § Path c (total effect): total effect of periodontal diseases on the outcomes without adjusting for mediators. ¶ Path c′ (natural direct effect): direct effect of periodontal diseases on the outcomes after accounting for each mediator. Solid lines represent statistically significant associations; dashed lines indicate non-significant associations. Line thickness reflects the relative magnitude of the proportion mediated. The line format between each mediator and its outcome specifically indicates whether the proportion mediated (indirect effect) is statistically significant. Line color represents the mediating effect of each mediator. MLTCs, multiple long-term conditions; Coeff, coefficient; CI, confidence interval, NHANES, national health and nutrition examination survey; CRP, C-reactive protein; ALT, alanine transaminase; AST, aspartate transaminase; WBC, white blood cells.

In UKB, systemic inflammatory biomarkers and liver enzymes may partially explain the relationship between periodontal inflammatory symptoms and MLTCs. The proportion of mediation included WBC (7.72%), neutrophils (7.62%), CRP (5.76%), ALT (1.30%), monocytes (1.25%), AST (0.36%), and platelet (0.28%) (Figure 3, Supplementary Materials: Table S7). Similar mediation patterns were observed in relation to the number of conditions. In NHANES, WBC (4.73%), neutrophils (5.61%), and CRP (4.70%) mediated the association between periodontitis and the number of conditions (Figure 3, Supplementary Materials: Table S7).

## 4. Discussion

In this large cross-sectional analysis of two population-based cohorts, we found consistent associations between oral inflammatory conditions and MLTCs across both the UK and US populations. Individuals with periodontal diseases had higher odds of having MLTCs, a greater number of chronic conditions, and more severe MLTCs burden. Mediation analyses suggested that systemic inflammatory biomarkers, particularly CRP levels, WBC and neutrophils, partially explained these associations. These findings contribute to a growing body of evidence suggesting that oral inflammatory symptoms are associated not only with poor oral health but may also be linked to disease clustering and greater chronic disease burden.

The prevalence of MLTCs observed in this study (approximately 50% in both UKB and NHANES) is consistent with estimates from high-income settings, highlighting the growing challenge of managing MLTCs in ageing populations [2]. Despite increasing recognition of the systemic impact of oral diseases, oral health remains largely absent from current MLTCs strategies and health policy frameworks. This omission represents a critical gap in chronic disease prevention and management.

Differences in MLTCs estimates across studies largely reflect the absence of a universally standardized case definition [16]. In this study, MLTCs were defined based on a list of 41 chronic conditions aligned with NICE guidelines and prior UKB analyses [18]. This approach supports comparability with existing UK-based research and ensures inclusion of clinically relevant chronic diseases.

Our findings align with previous evidence linking oral inflammatory diseases, particularly periodontitis to individual chronic conditions such as diabetes and cardiovascular diseases [7]. We extend these observations by examining the cumulative burden and severity of multimorbidity. The adjusted associations observed (UKB: OR 1.12; NHANES: OR 1.22) fall within the range reported by other studies, including longitudinal analyses using UKB data [10,32,33]. Differences in effect size likely reflect variability in exposure ascertainment (clinical vs. self-report) and differing definitions of systemic outcomes. The periodontal assessment methods used in the UK Biobank and NHANES were different and may affect the overall prevalence of periodontitis in each dataset. Although clinical examination is the standard method for assessing periodontal status, two systematic reviews have confirmed that self-reported periodontal assessment has acceptable validity, with overall sensitivity and specificity across questionnaire items ranging from 17% to 82% and 23% to 97% respectively in one study [34], and 4% to 93% and 58% to 94% respectively in another [14]. For each individual symptom used in UKB, loose teeth had a sensitivity of 27%, specificity of 90%, and diagnostic odds ratio of 2.35; bleeding gums had a sensitivity of 37%, specificity of 81%, and diagnostic odds ratio of 1.58; and painful gums had a sensitivity of 44%, specificity of 70%, and diagnostic odds ratio of 1.57 [34]. Due to the consistently low sensitivity values, the prevalence of periodontitis based on self-reported data is likely underestimated. If this misclassification is non-differential (equally distributed across participants with and without MLTCs), epidemiological theory predicts that it would bias the observed association towards the null, potentially explaining the more conservative odds ratio in UKB (OR 1.12) compared to NHANES (OR 1.22). However, we cannot exclude the possibility of differential misclassification, where underreporting of periodontal symptoms differs between those with and without MLTCs, in which case the direction of bias would be uncertain.

In addition, the variation in definitions of systemic outcomes might affect the variability in the effect size. The condition lists differed between datasets, with 41 conditions in UKB and 22 in NHANES, reflecting differences in data availability rather than arbitrary selection. Approximately 18 core conditions were represented in both datasets, encompassing the major contributors to multimorbidity burden including cardiovascular disease, diabetes, cancer, depression, and chronic respiratory conditions. The additional conditions unique to UKB were predominantly lower-prevalence disorders such as Ménière’s disease, bronchiectasis, multiple sclerosis, and endometriosis, which likely explains why MLTCs prevalence was virtually identical across both cohorts (UKB: 50.49%, NHANES: 50.25%) despite the difference in condition list size. The direct comparability of absolute prevalence estimates should be interpreted with caution, however, the consistency in the direction and magnitude of the association between periodontal diseases and MLTCs across both datasets supports the robustness of the findings.

Although the adjusted odds ratios observed in this study are modest at the individual level (UKB: OR 1.12; NHANES: OR 1.22), their clinical relevance becomes more apparent when examined alongside absolute differences in MLTCs prevalence. In UKB, MLTCs were present in 52.32% of participants reporting periodontal symptoms compared with 50.09% of those without, representing an absolute difference of 2.23 percentage points across a cohort of over 500,000 individuals. In NHANES (n = 10,714), this difference was more pronounced as MLTCs prevalence was 56.02% among those with clinically-assessed periodontitis compared with 46.03% among those without, an absolute difference of approximately 10 percentage points. The larger absolute difference in NHANES likely reflects the greater sensitivity of clinical periodontal assessment compared with self-report, and the higher overall burden of periodontitis in this population (42.26% vs. 17.80% in UKB). Considering the high prevalence of periodontal disease in both populations, these findings suggest that oral health represents a potentially modifiable correlate of multimorbidity burden at the population level.

Another important observation was that associations were stronger in participants aged ≤60 years, supporting the hypothesis that oral inflammation may be associated with earlier accumulation of chronic conditions, consistent with life-course models of multimorbidity [35]. The positive associations observed between periodontal status and both the number and severity of chronic conditions reinforce the concept of cumulative risk accumulation across the lifespan [36].

In NHANES, the stronger association observed for moderate rather than severe periodontitis may reflect several factors. First, survival and tooth loss bias may underrepresent the most severely affected individuals in the NHANES examination sample, as those with advanced disease are more likely to have undergone tooth extraction and thus may not meet criteria for full-mouth periodontal assessment. Second, the moderate periodontitis group was slightly older on average than the severe group, and age is independently associated with multimorbidity burden. Finally, residual confounding and heterogeneity within the severe category may have contributed to attenuating the observed effect.

Systemic inflammation is one of the well-established biological mechanisms linking periodontal disease to systemic conditions [8,24,37]. Periodontal inflammation has been proposed to trigger low-grade systemic inflammation and transient bacteraemia, promoting endothelial dysfunction, insulin resistance, and metabolic disruption [24]. In this analysis, restricting outcomes to inflammation-related MLTCs yielded similar effect estimates, and mediation analysis suggested that CRP, WBC, and neutrophils partially mediated these associations. Mediation appeared stronger in UKB than NHANES, possibly reflecting differences in sample characteristics, periodontal assessment method, or prevalence of comorbidities. Interestingly, liver enzymes (ALT and AST) also partially mediated associations in UKB, consistent with emerging evidence linking oral and hepatic inflammation through shared metabolic pathways [26]. However, given the cross-sectional design, the mediation findings should be interpreted as exploratory and hypothesis-generating rather than evidence of causal mechanisms. It is important to acknowledge that the proportion mediated by all assessed inflammatory mediators and liver enzymes was relatively small (0.25–7.72%), suggesting that inflammation is only one of several plausible pathways. Beyond inflammation, other plausible mechanisms include direct microbial dissemination, immune modulation, and shared behavioral or social determinants (e.g., smoking, deprivation, dietary habits) [24]. Importantly, an interventional study suggests that periodontal treatment reduces systemic inflammatory burden and improves vascular function, indicating that oral health interventions may modify long-term chronic disease trajectories [38].

Given the ageing population and rising MLTCs burden, the integration of oral health into MLTCs management frameworks is increasingly justified. If these associations are confirmed in longitudinal studies, lifestyle-based programmes targeting risk factors of highly prevalent chronic conditions could benefit from incorporating oral health promotion, particularly as part of multidisciplinary, patient-centered care models. Closer collaboration between medical and dental services—including joint screening, referral pathways, and patient education—could facilitate earlier detection and holistic management of multimorbidity.

### Strengths and Limitations

Several limitations must be acknowledged. First, periodontal status in UKB and some chronic conditions in both datasets were self-reported, which might introduce recall and misclassification biases. Among periodontal indicators, loose teeth had high specificity (92%) but low sensitivity (55%) [15]. This limitation was mitigated by combining multiple symptoms and adjusting for sociodemographic and behavioral confounders [39]. Second, reliance on self-report for medical diagnoses and periodontal symptoms may underestimate disease prevalence [39,40]; however, previous validation studies suggest moderate concordance between self-reports and clinical records [41]. Third, the cross-sectional nature of this analysis precludes conclusions about causality or temporal sequence. Fourth, the CMS was assessed by combining the original CMS and imputed version from a previous published study [18] for conditions without an officially validated weight. While this approach introduces some imprecision in individual condition weighting, the conditions without official weights were predominantly low-prevalence disorders whose contribution to the overall CMS distribution is limited. The categorization of CMS into quartiles for analysis reduces sensitivity to minor scoring inaccuracies compared to a continuous score approach. Nevertheless, the findings from severity analyses should be interpreted with this limitation in mind. Fifth, the absence of standardized MLTCs definitions limits comparability across studies. Sixth, despite adjustment for key socioeconomic indicators (Townsend deprivation index and household income in UKB; poverty income ratio in NHANES), ethnicity, smoking, BMI, and physical activity, residual confounding cannot be excluded. Notably, dietary quality, alcohol consumption, medication, and dental care access were not included as covariates. These unmeasured confounders should be considered when interpreting the findings, and future studies with more comprehensive covariate data are warranted. Lastly, the study findings might have been affected by selection bias as UKB recruited healthy volunteers (i.e., participants are systematically healthier, wealthier, and more educated than the general UK population), and it might underestimate the true prevalence of periodontal symptoms and MLTCs which would limit effect sizes. Further, the NHANES included only non-institutionalized participants. Therefore, older adults in care homes with the highest prevalence of multiple chronic conditions and periodontal burden are absent, particularly affecting the >60 age subgroup analyses.

Nonetheless, key strengths include the large sample size, cross-cohort replication using both self-reported and clinically assessed periodontal data, and the use of causal mediation analysis to explore underlying mechanisms. The combination of population-representative US data (NHANES) and UKB-based evidence supports external validity. Together, these findings add new insight into the interplay between oral health and chronic disease clustering.

## 5. Conclusions

Oral inflammatory diseases are independently associated with the presence, severity, and number of MLTCs. Systemic inflammation may be an important, but only partial, mediator of these associations. These findings suggest the potential benefit of integrating oral health into public health frameworks addressing multimorbidity and noncommunicable disease prevention. Given the shared risk factors and overlapping mechanisms, periodontal health promotion may represent a scalable and accessible strategy for reducing multimorbidity burden.

## Figures and Tables

**Table 1:** Characteristics of the study population in the UK Biobank dataset.

	Overall	No Periodontal Symptoms	Present Periodontal Symptoms	*p*-Value (No vs. Present Periodontal Symptoms)	Periodontal Symptoms		
					Painful Gum	Bleeding Gum	Loose Teeth
	500,612	411,521 (82.20%)	89,091 (17.80%)		15,221 (3.04%)	66,337 (13.25%)	21,687 (4.33%)
**Age (years), mean (SD)**	56.53 (8.09)	56.82 (8.10)	55.23 (7.95)	<0.001 ^†^	56.12 (8.02)	54.33 (7.87)	57.58 (7.49)
**Age group, n (%)**							
≤60 years old	308,141 (61.55%)	246,439 (59.88%)	61,702 (69.26)	<0.001 *	9835 (64.61%)	48,907 (73.73%)	12,727 (58.68%)
>60 years old	192,471 (38.45%)	165,082 (40.12%)	27,389 (30.74)		5386 (35.39%)	17,430 (26.27%)	8960 (41.32%)
**Gender, n (%)**							
Female	272,389 (54.41%)	219,268 (53.28%)	53,121 (59.63%)	<0.001 *	9550 (62.74%)	41,074 (61.92%)	10,948 (50.48%)
Male	228,223 (45.59%)	192,253 (46.72%)	35,970 (40.37%)		5671 (37.26%)	25,263 (38.08%)	10,739 (49.52%)
**Ethnicity, n (%)**							
White	471,452 (94.67%)	390,007 (95.30%)	81,445 (91.76%)	<0.001 *	13,490 (89.08%)	61,083 (92.39%)	19,205 (89.04%)
Mixed	2934 (0.59%)	2264 (0.55%)	670 (0.75%)		131 (0.87%)	506 (0.77%)	172 (0.80%)
Asian/Asian British	9650 (1.94%)	6925 (1.69%)	2725 (3.07%)		701 (4.63%)	1809 (2.74%)	876 (4.06%)
Black/Black British	7948 (1.60%)	5627 (1.37%)	2321 (2.61%)		458 (3.02%)	1703 (2.58%)	681 (3.16%)
Other	6034 (1.21%)	4433 (1.08%)	1601 (1.80%)		364 (2.40%)	1016 (1.54%)	635 (2.94%)
**Total household income, n (%)**							
<£18,000	96,735 (22.80%)	77,647 (22.33%)	19,088 (24.92%)	<0.001 *	4165 (32.75%)	12,497 (21.65%)	6292 (34.54%)
£18,000–£30,999	107,918 (25.43%)	88,596 (25.48%)	19,322 (25.23%)		3234 (25.43%)	14,162 (24.54%)	5024 (27.58%)
£31,000–£51,999	110,626 (26.07%)	90,763 (26.10%)	19,863 (25.93%)		2872 (22.58%)	15,796 (27.37%)	3984 (21.87%)
£52,000–£100,000	86,173 (20.31%)	71,259 (20.49%)	14,914 (19.47%)		2022 (15.90%)	12,371 (21.44%)	2458 (13.49%)
>£100,000	22,907 (5.40%)	19,504 (5.61%)	3403 (4.44%)		426 (3.35%)	2888 (5.00%)	460 (2.52%)
**Townsend deprivation index, mean (SD)**							
Least deprived	100,439 (20.09%)	85,134 (20.71%)	15,331 (17.24%)	<0.001 *	2359 (15.52%)	11,900 (17.97%)	3002 (13.86%)
Second least deprived	99,858 (19.98%)	83,894 (20.41%)	15,987 (17.97%)		2581 (16.98%)	12,254 (18.51%)	3413 (15.75%)
Middle	100,089 (20.02%)	83,122 (20.22%)	16,997 (19.11%)		2728 (17.95%)	13,052 (19.71%)	3746 (17.29%)
Second most deprived	99,901 (19.99%)	81,171 (19.75%)	18,760 (21.09%)		3223 (21.20%)	13,847 (20.91%)	4723 (21.80%)
Most deprived	99,565 (19.92%)	77,721 (18.91%)	21,871 (24.59%)		4309 (28.35%)	15,163 (22.90%)	6780 (31.30%)
**Educational level, n (%)**							
<High school	84,752 (17.76%)	70,011 (17.88%)	14,741 (17.21%)	<0.001 *	3207 (21.96%)	9116 (14.27%)	5126 (24.76%)
High school graduate	159,502 (33.42%)	129,218 (32.99%)	30,284 (35.35%)		4898 (33.53%)	22,979 (35.97%)	7204 (34.79%)
College degree or more	233,075 (48.83%)	192,436 (49.13%)	40,639 (47.44%)		6502 (44.51%)	31,797 (49.77%)	8375 (40.45%)
**Body Mass Index, mean (SD)**	27.43 (4.80)	27.33 (4.72)	27.88 (5.12)	<0.001 ^†^	27.87 (5.30)	27.87 (5.10)	28.34 (5.27)
**Smoking, n (%)**							
Current	52,681 (10.58%)	42,732 (10.45%)	9949 (11.21%)	<0.001 *	2285 (15.07%)	4910 (7.42%)	4885 (22.62%)
Previous	172,624 (34.67%)	139,709 (34.15%)	32,915 (37.07%)		5446 (35.93%)	24,177 (36.56%)	9142 (42.33%)
Never	272,561 (54.75%)	226,640 (55.40%)	45,921 (51.72%)		7428 (49.00%)	37,043 (56.02%)	7572 (35.06%)
**Marital status, living with partner, n (%)**							
Yes	362,061 (89.64%)	300,644 (90.27%)	61,417 (86.72%)	<0.001 *	9959 (85.13%)	46,575 (86.84%)	13,941 (85.57%)
No	41,829 (10.36%)	32,424 (9.73%)	9405 (13.28%)		1739 (14.87%)	7057 (13.16%)	2350 (14.43%)
**Physical activity, n (%)**							
High	245,858 (50.18%)	205,609 (51.04%)	40,249 (46.18%)	<0.001 *	6572 (44.07%)	30,056 (46.30%)	9791 (46.19%)
Low	244,103 (49.82%)	197,192 (48.96%)	46,911 (53.82%)		8342 (55.93%)	34,859 (53.70%)	11,405 (53.81%)
**Multiple Long-term conditions (MLTCs), n (%)**							
Yes	252,736 (50.49%)	206,122 (50.09%)	46,614 (52.32%)	<0.001 *	9533 (62.63%)	32,887 (49.58%)	12,733 (58.71%)
No	247,876 (49.51%)	205,399 (49.91%)	42,477 (47.68%)		5688 (37.37%)	33,450 (50.42%)	8954 (41.29%)
**Number of chronic conditions, n (%)**							
0–1 condition	247,876 (49.51%)	205,399 (49.91%)	42,477 (47.68%)	<0.001 *	5688 (37.37%)	33,450 (50.42%)	8954 (41.29%)
2 conditions	103,256 (20.63%)	84,976 (20.65%)	18,280 (20.52%)		3045 (20.01%)	13,538 (20.41%)	4531 (20.89%)
3 conditions	66,921 (13.37%)	54,742 (13.30%)	12,179 (13.67%)		2341 (15.38%)	8785 (13.24%)	3247 (14.97%)
4 or more conditions	82,559 (16.49%)	66,404 (16.14%)	16,155 (18.13%)		4147 (27.25%)	10,564 (15.92%)	4955 (22.85%)
**MLTCs Severity Score, mean (SD)**	0.78 (0.97)	0.81 (0.98)	0.77 (0.96)	<0.001 *	1.05 (1.14)	0.74 (0.92)	0.98 (1.09)
**MLTCs Severity Score, quartile, n (%)**							
Q1	127,641 (25.50%)	106,335 (25.84%)	21,306 (23.91%)	<0.001 *	2718 (17.86%)	16,964 (25.57%)	4294 (19.80%)
Q2	124,893 (24.95%)	102,722 (24.96%)	22,171 (24.89%)		3215 (21.12%)	17,346 (26.15%)	4753 (21.92%)
Q3	123,049 (24.58%)	100,224 (24.35%)	22,825 (25.62%)		4163 (27.35%)	16,744 (25.24%)	5747 (26.50%)
Q4	125,029 (24.98%)	102,240 (24.84%)	22,789 (25.58%)		5125 (33.67%)	15,283 (23.04%)	6893 (31.78%)
**Inflammation-related long-term conditions, n (%)**							
Yes	238,907 (47.72%)	195,036 (47.39%)	43,871 (49.24%)	<0.001 *	8923 (58.62%)	30,912 (46.60%)	12,111 (55.84%)
No	261,705 (52.28%)	216,485 (52.61%)	45,220 (50.76%)		6298 (41.38%)	35,425 (53.40%)	9576 (44.16%)
**Mediators, mean (SD)**							
White blood cells count (10^9^ cells/L)	6.88 (2.12)	6.85 (2.04)	7.05 (2.47)	<0.001 ^‡^	7.13 (3.82)	6.96 (2.05)	7.43 (2.11)
Platelet count (10^9^ cells/L)	252.99 (60.05)	252.30 (59.77)	256.11 (61.22)	0.006 ^‡^	257.41 (62.36)	256.61 (60.96)	254.71 (62.89)
C-Reactive Protein (mg/L)	2.60 (4.36)	2.55 (4.33)	2.81 (4.50)	<0.001 ^‡^	3.14 (5.04)	2.67 (4.24)	3.34 (5.16)
Lymphocyte count (10^9^ cells/L)	1.97 (1.17)	1.95 (1.13)	2.01 (1.37)	<0.001 ^‡^	2.05 (1.97)	1.99 (1.21)	2.10 (0.96)
Monocyte count (10^9^ cells/L)	0.48 (0.27)	0.48 (0.22)	0.48 (0.44)	<0.001 ^‡^	0.48 (0.96)	0.47 (0.20)	0.50 (0.25)
Neutrophils count (10^9^ cells/L)	4.23 (1.42)	4.20 (1.41)	4.34 (1.47)	<0.001 ^‡^	4.37 (1.65)	4.28 (1.40)	4.60 (1.59)
Alanine aminotransferase (U/L)	23.55 (14.18)	23.46 (13.93)	23.92 (15.28)	<0.001 ^‡^	23.55 (14.46)	23.99 (15.57)	24.27 (15.08)
Aspartate aminotransferase (U/L)	26.23 (10.66)	26.23 (10.47)	26.21 (11.48)	0.164 ^‡^	26.19 (10.83)	26.15 (11.71)	26.62 (11.53)

* Pearson’s chi-square tests for categorical variables, ^†^ *t*-tests, and ^‡^ adjusted *t*-test (adjusted for age, gender, education level, ethnicity, smoking, and BMI). SD = standard deviation.

**Table 2:** Characteristics of the study population in the NHANES dataset.

	Overall (n = 10,714)	No Periodontitis (n = 5231) 57.75% (1.381)	Total Periodontitis (n = 5483) 42.26% (1.381)	*p*-Value (No vs. Present Periodontitis)	Mild Periodontitis (n = 490) 4.30% (0.347)	Moderate Periodontitis (n = 3794) 30.1% (1.12)	Severe Periodontitis (n = 1199) 7.86% (0.518)
**Age, mean (SE)**	50.84 (0.24)	48.085 (0.28)	54.597 (0.34)	<0.001 ^†^	47.407 (0.65)	55.604 (0.41)	54.672 (0.46)
**Age group, % (SE)**							
≤60 years old	75.44 (0.60)	47.26 (1.16)	28.18 (0.95)	<0.001 *	3.67 (0.31)	18.98 (0.73)	5.54 (0.36)
>60 years old	24.56 (0.60)	10.49 (0.52)	14.08 (0.65)		0.63 (0.10)	11.13 (0.60)	2.32 (0.25)
**Gender, % (SE)**							
Male	48.83 (0.51)	24.28 (0.80)	24.55 (0.70)	<0.001 *	2.46 (0.23)	16.46 (0.58)	5.64 (0.38)
Female	51.17 (0.51)	33.46 (0.86)	17.71 (0.78)		1.84 (0.19)	13.65 (0.66)	2.22 (0.21)
**Ethnicity, % (SE)**							
Mexican American	8.09 (1.09)	3.26 (0.39)	4.83 (0.72)	<0.001 *	0.52 (0.09)	3.24 (0.51)	1.08 (0.20)
Other Hispanic	5.44 (0.71)	2.80 (0.34)	2.63 (0.39)		0.35 (0.08)	1.87 (0.28)	0.42 (0.07)
Non-Hispanic White	68.39 (1.94)	43.06 (1.68)	25.33 (1.37)		2.63 (0.31)	18.59 (1.10)	4.11 (0.44)
Non-Hispanic Black	10.70 (0.91)	4.65 (0.37)	6.05 (0.63)		0.59 (0.08)	3.90 (0.40)	1.57 (0.21)
Other Race—Including Multi-Racial	7.39 (0.56)	3.98 (0.33)	3.41 (0.35)		0.22 (0.04)	2.51 (0.26)	0.69 (0.12)
**Family Poverty Level, % (SE)**							
<100	11.23 (0.69)	4.43 (0.29)	6.8 (0.50)	<0.001 *	0.59 (0.10)	4.61 (0.39)	1.60 (0.16)
100–199	17.77 (0.73)	8.26 (0.49)	9.51 (0.52)		0.81 (0.10)	6.56 (0.42)	2.14 (0.22)
200–399	26.82 (1.06)	14.86 (0.84)	11.96 (0.67)		1.24 (0.16)	8.78 (0.51)	1.94 (0.23)
>400	44.19 (1.46)	30.20 (1.36)	13.99 (0.61)		1.65 (0.19)	10.16 (0.47)	2.18 (0.25)
**Acculturation Score, % (SE)**							
0: Foreign born + live in US <10 years	3.794 (0.40)	1.769 (0.21)	2.025 (0.24)	<0.001 *	0.223 (0.05)	1.443 (0.18)	0.359 (0.07)
1: Foreign born + live in US 10–19 years	5.317 (0.40)	2.65 (0.22)	2.667 (0.23)		0.309 (0.06)	1.852 (0.18)	0.506 (0.07)
2: Foreign born + live in US ≥20 years	9.221 (0.63)	4.565 (0.32)	4.657 (0.40)		0.297 (0.05)	3.174 (0.27)	1.186 (0.15)
3: US born	81.67 (1.22)	48.91 (1.48)	32.76 (1.39)		3.485 (0.32)	23.49 (1.145)	5.788 (0.44)
**Acculturation Score, mean (SE)**	2.69 (0.02)	2.74 (0.02)	2.62 (0.03)	<0.001 ^†^	2.63 (0.05)	2.63 (0.03)	2.58 (0.04)
**Education Level, % (SE)**							
<High School	15.32 (0.87)	5.40 (0.38)	9.93 (0.60)	<0.001 *	0.63 (0.08)	6.77 (0.46)	2.53 (0.23)
High School	20.82 (0.78)	9.87 (0.51)	10.96 (0.55)		1.16 (0.12)	7.46 (0.47)	2.34 (0.20)
College or Above	63.78 (1.27)	42.46 (1.50)	21.34 (0.70)		2.51 (0.27)	15.82 (0.60)	3.00 (0.28)
**Body Mass Index, mean (SE)**	29.17 (0.11)	28.94 (0.13)	29.50 (0.15)	0.002 ^†^	30.35 (0.45)	29.48 (0.18)	29.09 (0.25)
**Smoking status, % (SE)**							
Smoking	17.41 (0.50)	6.53 (0.33)	10.88 (0.46)	<0.001 *	0.71 (0.10)	7.19 (0.39)	2.98 (0.23)
Non-smoking	82.59 (0.50)	51.21 (1.28)	31.38 (1.01)		3.58 (0.31)	22.91 (0.91)	4.88 (0.35)
**Marital status, % (SE)**							
Married	63.27 (0.87)	39.39 (1.30)	23.89 (0.82)	<0.001 *	2.59 (0.23)	17.20 (0.73)	4.10 (0.33)
Widowed	5.32 (0.23)	2.18 (0.19)	3.14 (0.19)		0.14 (0.03)	2.48 (0.16)	0.51 (0.07)
Divorced/Separated	14.94 (0.43)	7.50 (0.29)	7.44 (0.41)		0.66 (0.11)	5.11 (0.34)	1.67 (0.17)
Never Married	10.31 (0.52)	5.68 (0.37)	4.63 (0.31)		0.62 (0.08)	3.12 (0.20)	0.89 (0.12)
Living with Partner	6.10 (0.39)	2.97 (0.25)	3.13 (0.27)		0.29 (0.05)	2.15 (0.22)	0.70 (0.10)
**Multiple Long-term conditions (MLTCs), % (SE)**							
Yes	50.25 (0.85)	26.58 (0.66)	23.67 (0.88)	<0.001 *	2.03 (0.18)	17.5 (0.80)	4.14 (0.33)
No	49.75 (0.85)	31.16 (1.02)	18.59 (0.74)		2.27 (0.25)	12.6 (0.51)	3.72 (0.27)
**Number of conditions, % (SE)**							
0 and 1 condition	49.75 (0.85)	31.16 (1.02)	18.59 (0.74)	<0.001 *	2.27 (0.25)	12.60 (0.51)	3.72 (0.27)
2 conditions	23.83 (0.57)	13.65 (0.49)	10.17 (0.43)		1.00 (0.10)	7.17 (0.38)	2.00 (0.18)
3 conditions	13.05 (0.54)	6.51 (0.40)	6.54 (0.39)		0.64 (0.10)	4.90 (0.33)	0.99 (0.14)
4 or more conditions	13.37 (0.55)	6.41 (0.32)	6.96 (0.43)		0.38 (0.08)	5.43 (0.35)	1.15 (0.12)
**MLTCs Severity Score, mean (SE)**	0.55 (0.01)	0.48 (0.01)	0.66 (0.02)	<0.001 ^†^	0.43 (0.04)	0.72 (0.02)	0.57 (0.02)
**MLTCs Severity Score Quartile, % (SE)**							
Quartile 1	41.24 (0.81)	26.33 (0.92)	14.91 (0.64)	<0.001 *	1.89 (0.23)	10.12 (0.46)	2.90 (0.20)
Quartile 2	15.43 (0.49)	9.16 (0.42)	6.27 (0.29)		0.67 (0.12)	4.26 (0.26)	1.34 (0.17)
Quartile 3	20.98 (0.59)	11.06 (0.45)	9.92 (0.46)		0.95 (0.11)	7.06 (0.36)	1.92 (0.17)
Quartile 4	22.35 (0.50)	11.19 (0.45)	11.16 (0.53)		0.79 (0.13)	8.66 (0.50)	1.71 (0.17)
**Inflammation-related long-term conditions, % (SE)**							
Yes	47.87 (0.81)	25.50 (0.65)	22.37 (0.83)	<0.001 *	1.89 (0.16)	16.74 (0.76)	3.73 (0.31)
No	52.13 (0.81)	32.34 (1.04)	19.89 (0.77)		2.40 (0.25)	13.36 (0.54)	4.13 (0.29)
**Mediators, mean (SE)**							
White blood cell (1000 cells/µL)	7.09 (0.04)	6.92 (0.04)	7.33 (0.05)	<0.001 ^‡^	7.21 (0.11)	7.26 (0.07)	7.67 (0.10)
Platelet (1000 cells/µL)	237.24 (0.96)	238.03 (0.10)	236.14 (1.53)	0.801 ^‡^	244.82 (4.18)	235.13 (1.73)	235.26 (3.14)
C-reactive protein (mg/dL)	0.36 (0.02)	0.33 (0.02)	0.40 (0.03)	0.028 ^‡^	0.47 (0.10)	0.37 (0.03)	0.44 (0.04)
Lymphocyte (1000 cells/µL)	2.07 (0.01)	2.04 (0.02)	2.11 (0.01)	0.755 ^‡^	2.13 (0.04)	2.10 (0.02)	2.13 (0.03)
Monocyte (1000 cells/µL)	0.55 (0.004)	0.53 (0.004)	0.57 (0.007)	0.002 ^‡^	0.54 (0.011)	0.56 (0.005)	0.60 (0.019)
Neutrophil (1000 cells/µL)	4.24 (0.03)	4.12 (0.03)	4.40 (0.04)	<0.001 ^‡^	4.29 (0.08)	4.34 (0.05)	4.67 (0.08)
Alanine aminotransferase (U/L)	26.06 (0.21)	25.45 (0.26)	26.89 (0.39)	0.672 ^‡^	27.92 (0.94)	26.37 (0.46)	28.36 (0.97)
Aspartate aminotransferase (U/L)	26.23 (0.18)	25.77 (0.26)	26.87 (0.31)	0.737 ^‡^	26.36 (0.69)	26.52 (0.37)	28.50 (0.81)

* Pearson’s chi-square tests for categorical variables, ^†^ survey-weighted linear regression models, and ^‡^ adjusted *t*-test (adjusted for age, gender, education level, ethnicity, smoking, and BMI). SE = standard error.

## Data Availability

All data generated or analyzed during this study are included in this article, and its Supplementary Material files. Further enquiries can be directed to the corresponding author.

## Supplementary Materials

The following supporting information can be downloaded at: https://www.mdpi.com/article/10.3390/jcm15114029/s1, Table S1: UK Biobank codes for chronic conditions (Ronaldson et al, 2022) [18]; Table S2: NHANES assessment for chronic conditions; Table S3: Severity weights score from the Cambridge Multimorbidity Score and imputed weight (Ronaldson et al, 2022) [18]; Table S4: Missing data in each variable; Table S5: Association between periodontal disease and MLTCs: UKB and NHANES full models; Table S6: Association between periodontal diseases and MLTCs; Table S7: Mediation analyses for the association between periodontal diseases and MLTCs/ number of chronic conditions.

## Abbreviations

Alanine transaminase (ALT); Aspartate transaminase (AST); Body mass index (BMI); Clinical attachment level (CAL); Centers for Disease Control and Prevention (CDC); Controlled direct effects (CDE); Confidence interval (CI); Cambridge Multimorbidity Score (CMS); C-reactive protein (CRP); Multiple imputation by chained equations (MICE); Multiple long-term conditions (MLTCs); Marginal total effects (MTE); Natural direct effects (NDE); National Health and Nutrition Examination Survey (NHANES); National Institute for Health and Care Excellence (NICE); Natural indirect effects (NIE); Odds ratio (OR); Probing pocket depth (PPD); Standard error (SE); UK Biobank (UKB).
