# Supplementary material for: Oral Health, Inflammation, and the Burden of Multiple Long-Term Conditions: Cross-Sectional Analyses from UK Biobank and NHANES
Source: J Clin Med. 2026 May 22;15(11):4029. doi: 10.3390/jcm15114029 (PMC13258311; doi:10.3390/jcm15114029)
Supplement: Supplementary file 1 [file jcm-15-04029-s001.zip › jcm-4282373-supplementary.pdf]

Supplementary material

Table S1 UK Biobank codes for chronic conditions (Ronaldson et al, 2022)

Conditions	Self-reported (Data field 20002)	ICD-10 (Data field 41270)
Alcohol problems and other psychoactive substance abuse	1408 Alcohol dependency<br>1604 Alcoholic liver disease/alcoholic cirrhosis<br>1409 Opioid dependency<br>1410 Other substance abuse/dependency	F10 Mental and behavioural disorders due to use of alcohol<br>F11 Mental and behavioural disorders due to use of opioids<br>F12 Mental and behavioural disorders due to use of cannabinoids<br>F13 Mental and behavioural disorders due to use of sedatives or hypnotics<br>F14 Mental and behavioural disorders due to use of cocaine<br>F15 Mental and behavioural disorders due to use of other stimulants, including caffeine<br>F16 Mental and behavioural disorders due to use of hallucinogens<br>F19 Mental and behavioural disorders due to multiple drug use and use of other psychoactive substances
Anorexia or bulimia	1470 Anorexia/bulimia/other eating disorder	F50 Eating disorders
Anxiety	1287 Anxiety/panic attacks<br>1615 Obsessive compulsive disorder (OCD)<br>1469 Post-traumatic stress disorder (PTSD)<br>1614 Stress	F41 Other anxiety disorders<br>F42 Obsessive-compulsive disorder<br>F431 Post-traumatic stress disorder
Asthma	1111 Asthma	J45 Asthma
Atrial fibrillation	1471 Atrial fibrillation	I48 Atrial fibrillation and flutter
		I49 Other cardiac arrhythmias
Bronchiectasis	1114 Bronchiectasis	J47 Bronchiectasis
Cancer	UK Biobank data field 2453	C17 Malignant neoplasm of small intestine
		C18 Malignant neoplasm of colon
		C19 Malignant neoplasm of rectosigmoid junction
		C20 Malignant neoplasm of rectum
		C21 Malignant neoplasm of anus and anal canal
		C16 Malignant neoplasm of stomach
		C15 Malignant neoplasm of oesophagus
		C22 Malignant neoplasm of liver and intrahepatic bile ducts
		C25 Malignant neoplasm of pancreas
		C50 Malignant neoplasm of breast
		C34 Malignant neoplasm of bronchus and lung
		C40 Malignant neoplasm of bone and articular cartilage of limbs
		C41 Malignant neoplasm of bone and articular cartilage of other and unspecified sites
		C43 Malignant melanoma of skin
		C71 Malignant neoplasm of brain
		C53 Malignant neoplasm of cervix uteri
		C55 Malignant neoplasm of uterus, part unspecified
		C56 Malignant neoplasm of ovary
		C61 Malignant neoplasm of prostate
		C64 Malignant neoplasm of kidney, except renal pelvis
		C80 Malignant neoplasm without specification of site

		C81 Lymphocyte-rich classical Hodgkin lymphoma
		C82 Follicular lymphoma
		C83 Non-follicular lymphoma
		C84 Mature T/NK-cell lymphomas
		C85 Other specified and unspecified types of non-Hodgkin lymphoma
		C86 Other specified types of T/NK-cell lymphoma
		C88 Malignant immunoproliferative diseases and certain other B-cell lymphomas
		C90 Multiple myeloma and malignant plasma cell neoplasms
		C91 Lymphoid leukaemia
		C92 Myeloid leukaemia
		C93 Monocytic leukaemia
		C94 Other leukaemia's of specified cell type
		C95 Leukaemia of unspecified cell type
		C96 Other and unspecified malignant neoplasms of lymphoid, hematopoietic and related tissue
		C00 Malignant neoplasm of lip
		C01 Malignant neoplasm of base of tongue
		C02 Malignant neoplasm of other and unspecified parts of tongue
		C03 Malignant neoplasm of gum
		C04 Malignant neoplasm of floor of mouth
		C05 Malignant neoplasm of palate
		C06 Malignant neoplasm of other and unspecified parts of mouth
Chronic fatigue syndrome (CFS)	1482 Chronic fatigue syndrome	R53.82 Chronic fatigue, unspecified
Chronic kidney disease (CKD)	1192 Renal/kidney failure	N17 Acute kidney failure
	1193 Renal failure requiring dialysis	N18 Chronic kidney disease (CKD)
	1194 Renal failure not requiring dialysis	N19 Unspecified kidney failure
	1427 Polycystic kidney	N00 Acute nephritic syndrome
	1519 Kidney nephropathy	N01 Rapidly progressive nephritic syndrome
	1520 IGA nephropathy	N03 Chronic nephritic syndrome
	1607 Diabetic nephropathy	N04 Nephrotic syndrome
		N05 Unspecified nephritic syndrome
		N08 Glomerular disorders in diseases classified elsewhere
		E08.22 Diabetes mellitus due to underlying condition with diabetic chronic kidney disease
		E09.22 Drug or chemical induced diabetes mellitus with diabetic chronic kidney disease
		E10.22 Type 1 diabetes mellitus with diabetic chronic kidney disease
		E11.22 Type 2 diabetes mellitus with diabetic chronic kidney disease
		E13.22 Other specified diabetes mellitus with diabetic chronic kidney disease
		I12 Hypertensive chronic kidney disease
		I13 Hypertensive heart and chronic kidney disease
Chronic obstructive pulmonary disorder (COPD)	1112 Chronic obstructive airways disease/COPD	J44 Other chronic obstructive pulmonary disease
	1113 Emphysema/chronic bronchitis	J43 Emphysema
	1472 Emphysema	J42 Unspecified chronic bronchitis
		J41 Simple and mucopurulent chronic bronchitis

		J40 Bronchitis, not specified as acute or chronic
Chronic sinusitis	1416 Chronic sinusitis	J32 Chronic sinusitis
Connective tissue disorders	1322 Myositis/myopathy	K90 Intestinal malabsorption/coeliac disease
	1373 Connective tissue disorder	M05 Rheumatoid arthritis
	1377 Polymyalgia rheumatica	M06 Other rheumatoid arthritis
	1381 Systemic lupus erythematosis/SLE	M07 Psoriatic and enteropathic arthropathies
	1382 Sjogren's syndrome/sicca syndrome	M08 Juvenile arthritis
	1383 Dermatopolymyositis	M30 Polyarteritis nodosa and related conditions
	1384 Scleroderma/systemic sclerosis	M31 Other necrotizing vasculopathies
	1456 Malabsorption/coeliac disease	M32 Systemic lupus erythematosus
	1464 Rheumatoid arthritis	M33 Dermatopolymyositis
	1477 Psoriatic arthropathy	M34 Systemic sclerosis
	1480 Dermatomyositis	M35 Other systemic involvement of connective tissue
	1481 Polymyositis	M36 Systemic disorders of connective tissue in diseases classified elsewhere
		M60 Myositis
Coronary heart disease (CHD)	1074 Angina	I20 Angina pectoris
	1075 Heart attack/myocardial infarction	I21 Acute myocardial infarction
		I22 Subsequent myocardial infarction
		I23 Certain current complications following acute myocardial infarction
		I24 Other acute ischaemic heart diseases
		I25 Chronic ischaemic heart disease
Dementia	1263 Dementia/Alzheimer's/ cognitive impairment	F01 Vascular dementia
		F02 Dementia in other diseases classified elsewhere
		F03 Unspecified dementia
		G30 Alzheimer disease
		G31.83 Dementia with Lewy bodies
		G31.0 Circumscribed brain atrophy
		G31.1 Senile degeneration of brain, not elsewhere classified
		G31.01 Pick's disease
		G31.09 Other frontotemporal dementia
Diabetes	1220 Diabetes	E11 Non-insulin-dependent diabetes mellitus
	1222 Type 1 diabetes	E10 Insulin-dependent diabetes mellitus
	1223 Type 2 diabetes	E09 Drug or chemical induced diabetes mellitus
	1276 Diabetic eye disease	E08 Diabetes mellitus due to underlying condition
	1468 Diabetic neuropathy/ulcers	E13 Other specified diabetes mellitus
	1607 Diabetic nephropathy	O24.4 Gestational diabetes
Diverticular disease	1458 Diverticular disease/diverticulitis	K57 Diverticular disease of intestine
Dyspepsia/ulcer	1138 Gastro-oesophageal reflux/gastric reflux	K21 Gastro-oesophageal reflux disease
	1139 Oesophagitis/barretts oesophagus	K22 Other diseases of oesophagus
	1142 Gastric/stomach ulcers	K25 Gastric ulcer
	1143 Gastritis/gastric erosions	K26 Duodenal ulcer
	1442 Helicobacter pylori	K27 Peptic ulcer, site unspecified
	1457 Duodenal ulcer	K28 Gastrojejunal ulcer
	1474 Hiatus hernia	K29 Gastritis and duodenitis
	1510 Dyspepsia/indigestion	K30 Dyspepsia
Endometriosis	1402 Endometriosis	N80 Endometriosis

Epilepsy	1264 Epilepsy	G40 Epilepsy
Glaucoma	1277 Glaucoma	H40 Glaucoma
		H42 Glaucoma in diseases classified elsewhere
Heart failure	1076 Heart failure/pulmonary oedema	I46 Cardiac arrest
	1079 Cardiomyopathy	I50 Heart failure
	1588 Hypertrophic cardiomyopathy	
Hepatitis	1156 Infective/viral hepatitis	B15 Acute hepatitis A
	1578 hepatitis A	B16 Acute hepatitis B
	1579 hepatitis B	B17 Other acute viral hepatitis
	1580 Hepatitis C	B18 Chronic viral hepatitis
	1581 Hepatitis D	B19 Unspecified viral hepatitis
	1582 Hepatitis E	
Hypertension	1065 Hypertension	I10 Essential (primary) hypertension
	1072 Essential hypertension	I11 Hypertensive heart disease
		I12 Hypertensive renal disease
		I13 Hypertensive heart and renal disease
		I15 Secondary hypertension
Inflammatory bowel disease (IBD)	1461 Inflammatory bowel disease	K50 Crohn disease [regional enteritis]
	1462 Crohn’s disease	K51 Ulcerative colitis
	1463 Ulcerative colitis	K52 Other noninfective gastroenteritis and colitis
Irritable bowel syndrome (IBS)	1154 Irritable bowel syndrome	K58 Irritable bowel syndrome
Liver disease	1141 Oesophageal varicies	K70 Alcoholic liver disease
	1157 Non-infective hepatitis	K71 Toxic liver disease
	1158 Liver failure/cirrhosis	K72 Hepatic failure, not elsewhere classified
	1506 Primary biliary cirrhosis	K73 Chronic hepatitis, not elsewhere classified
		K74 Fibrosis and cirrhosis of liver
		K75 Other inflammatory liver diseases
		K76 Other diseases of liver
		K77 Liver disorders in diseases classified elsewhere
Méniére’s disease	1421 Méniére disease	H81.0 Méniére disease
Migraine	1265 Migraine	G43 Migraine
Multiple sclerosis (MS)	1261 Multiple sclerosis	G35 Multiple sclerosis
Osteoporosis	1309 Osteoporosis	M80 Osteoporosis with pathological fracture
		M81 Osteoporosis without pathological fracture
		M82 Osteoporosis in diseases classified elsewhere
Painful conditions	1257 Trapped nerve/compressed nerve	M45 Ankylosing spondylitis
	1294 Back problem	M46 Other inflammatory spondylopathies
	1311 Spine arthritis/spondylitis	M47 Spondylosis
	1312 Prolapsed disc/slipped disc	M48 Other spondylopathies
	1313 Ankylosing spondylitis	M49 Spondylopathies in diseases classified elsewhere
	1436 Headaches (not migraine)	M50 Cervical disc disorders
	1465 Osteoarthritis	M51 Other intervertebral disc disorders
	1466 Gout	M53 Other dorsopathies, not elsewhere classified
	1476 Sciatica	M54 Dorsalgia
	1478 Cervical spondylosis	R51 Headache
	1523 Trigeminal neuralgia	G44 Other headache syndromes
	1532 Disc problem	M10 Gout
	1533 Disc degeneration	M11 Other crystal arthropathies
	1534 Back pain	M12 Other specific arthropathies
	1537 Joint pain	M13 Other arthritis
	1538 Arthritis	M14 Arthropathies in other diseases classified elsewhere
	1540 Plantar fasciitis	M72.2 Plantar fasciitis

	1541 Carpal tunnel syndrome	G50.0 Trigeminal neuralgia
	1542 Fibromyalgia	G50.1 Atypical facial pain
	1573 Shingles	G56.0 Carpal tunnel syndrome
		M79.7 Fibromyalgia
		B02 Herpes zoster (shingles)
Parkinson's disease	1262 Parkinson's disease	G20 Parkinson's disease
		G21 Secondary parkinsonism
Peripheral vascular disease (PVD)	1067 Peripheral vascular disease	I700 Atherosclerosis of aorta
	1087 Leg claudication/intermittent claudication	I702 Atherosclerosis of arteries of extremities
		I708 Atherosclerosis of other arteries
		I709 Generalized and unspecified atherosclerosis
		I731 Thromboangiitis obliterans [Buerger]
		I738 Other specified peripheral vascular diseases
		I739 Peripheral vascular disease, unspecified
Pernicious anaemia	1331 Pernicious anaemia	D51.0 Vitamin B12 deficiency anaemia due to intrinsic factor deficiency
Polycystic ovarian syndrome (PCOS)	1350 Polycystic ovarian syndrome	E28.2 Polycystic ovarian syndrome
Prostate conditions (not cancer)	1207 Prostate problem (not cancer)	N40 Hyperplasia of prostate
	1396 Enlarged prostate	N41 Inflammatory diseases of prostate
	1516 Benign prostatic hypertrophy	N42 Other disorders of prostate
Psoriasis/eczema	1452 Eczema/dermatitis	L20 Atopic dermatitis
	1453 Psoriasis	L21 Seborrhoeic dermatitis
		L22 Diaper [napkin] dermatitis
		L23 Allergic contact dermatitis
		L24 Irritant contact dermatitis
		L25 Unspecified contact dermatitis
		L26 Exfoliative dermatitis
		L27 Dermatitis due to substances taken internally
		L28 Lichen simplex chronicus and prurigo
		L29 Pruritis
		L30 Other and unspecified dermatitis
		L40 Psoriasis
		L41 Parapsoriasis
Stroke/transient ischaemic attack (TIA)	1081 Stroke	I60 Subarachnoid haemorrhage
	1082 Transient ischaemic attack	I61 Intracerebral haemorrhage
	1086 Subarachnoid haemorrhage	I62 Other nontraumatic intracranial haemorrhage
	1491 Brain haemorrhage	I63 Cerebral infarction
	1583 Ischaemic stroke	I65 Occlusion and stenosis of precerebral arteries, not resulting in cerebral infarction
		I66 Occlusion and stenosis of cerebral arteries, not resulting in cerebral infarction
		I67 Other cerebrovascular diseases
		I68 Cerebrovascular disorders in diseases classified elsewhere
		I69 Sequelae of cerebrovascular disease
Thyroid conditions	1224 Thyroid problem (not cancer)	E00 Congenital iodine-deficiency syndrome
	1225 Hyperthyroidism/thyrotoxicosis	E01 Iodine-deficiency-related thyroid disorders and allied conditions
	1226 Hypothyroidism/myxoedema	E02 Subclinical iodine-deficiency hypothyroidism
	1428 Thyroiditis	E03 Other hypothyroidism
	1522 Grave's disease	E04 Other nontoxic goitre
	1610 Thyroid goitre	E05 Thyrotoxicosis [hyperthyroidism]
		E06 Thyroiditis
		E07 Other disorders of thyroid

Table S2 NHANES assessment for chronic conditions

Conditions	Self-reported data	Examination/ laboratory data
Anaemia	MCQ053 – Taking treatment for anaemia/past 3 months	NA
Arthritis	MCQ160A – Doctor ever said you had arthritis	NA
Asthma	MCQ010 – Ever been told you have asthma	NA
Bronchitis	MCQ160K – Ever told you had chronic bronchitis	NA
Cancer	MCQ220 – Ever told you had cancer or malignancy	NA
Celiac disease	MCQ082 – Ever been told you have celiac disease	NA
Combined heart disease	MCQ160B – Ever told had congestive heart failure<br>MCQ160C – Ever told you had coronary heart disease	NA
Depression	DPQ010/ DPQ020/ DPQ030/ DPQ040/ DPQ050/ DPQ060/ DPQ070/ DPQ080 / DPQ090: assessed with the Patient Health Questionnaire – PHQ-9; participants had depressive symptoms whenever PHQ-9 ≥ 10	NA
Diabetes	DIQ010	LBXGH – glycated haemoglobin (HbA1c) ≥6.5%<br>LBXGLT – serum glucose at 2 hours following a 75 g glucose load (OGTT) ≥200 mg/dL<br>LBXGLU – fasting plasma glucose ≥126 mg/dL
Emphysema	MCQ160G – Ever told you had emphysema	NA
Gout	MCQ160N – Doctor ever told you that you had gout	NA
Hypercholesterolemia	BPQ080 – Doctor told you - high cholesterol level	LBXTR – Triglyceride ≥150 mg/dL<br>LBDLDL – LDL-cholesterol ≥130 mg/dL<br>LBDHDD – Direct HDL-Cholesterol <40 mg/dL (women), <50 mg/dL (men)<br>LBXTC – Total Cholesterol ≥200 mg/dL
Hypertension	NA	No hypertension – measured Systolic Blood Pressure (SBP) < 120 mmHg and measured Diastolic Blood Pressure (DBP) < 80mmHg<br>Borderline hypertension – 120≤SBP<140 mmHg or 80≤DBP<90 mmHg<br>Hypertension – average of SBP≥140mmHg or DBP≥90mmHg
Jaundice	MCQ203 – Ever been told you have jaundice? (2013-14)	NA
Kidney disease	KIQ022 - Ever told you had weak/failing kidneys	NA
Liver disease	MCQ160L – Ever told you had any liver condition	NA
Memory cognitive disorder	MCQ084 – Difficulties in thinking or remembering (2011-12, 2013-14)	NA
Osteoporosis	NA	DXXOFBMD – Total femur BMD<br>DXXNKBMD – Femoral neck BMD<br>Osteoporosis – T-score < -2.5<br>Osteopenia – T-score -1 to -2.5<br>Normal – T-score >-1
Psoriasis	MCQ070 – Ever been told you have psoriasis?	NA
Stroke	MCQ160F – Ever told you had a stroke	NA
Thyroid problems	MCQ160M – Ever told you had a thyroid problem	NA

Vision	MCQ140 – Trouble seeing even with glass/contacts	NA

Table S3 Severity weights score from the Cambridge Multimorbidity Score and imputed weight (Ronaldson et al, 2022)

**Conditions**	**Severity weight**
**UK Biobank**	
Alcohol problems and other psychoactive substance abuse	0.47
Anorexia or bulimia	0.34
Anxiety	0.47
Asthma	0.18
Atrial fibrillation	1.3
Bronchiectasis	0.66
Cancer	1.5
Chronic fatigue syndrome (CFS)	-0.1
Chronic kidney disease (CKD)	0.51
Chronic obstructive pulmonary disorder (COPD)	1.41
Chronic sinusitis	0.13
Connective tissue disorders	0.4
Coronary heart disease (CHD)	0.46
Dementia	2.46
Depression	0.47
Diabetes	0.71
Diverticular disease	-0.02
Dyspepsia/ulcer	0.2
Endometriosis	0.05
Epilepsy	0.85
Glaucoma	0.44
Heart failure	1.12
Hepatitis	0.67
Hypertension	0.09
Inflammatory bowel disease (IBD)	0.44
Irritable bowel syndrome (IBS)	0.18
Liver disease	0.72
Meniere disease	0.36
Migraine	0.07
Multiple sclerosis (MS)	0.69
Osteoporosis	0.76
Painful conditions	0.87
Parkinson’s disease	1.29
Peripheral vascular disease (PVD)	0.53
Pernicious anaemia	0.97
Polycystic ovarian syndrome (PCOS)	-1.02
Prostate conditions (not cancer)	0.01
Psoriasis/eczema	0.25
Severe mental illness	0.58
Stroke/transient ischaemic attack (TIA)	0.77

Thyroid conditions	0.08
**NHANES**	
Anaemia	0.97
Arthritis	NA
Asthma	0.18
Bronchitis	NA
Cancer	1.5
Celiac disease	NA
Combined heart disease	0.79
Depression	0.47
Diabetes	0.71
Emphysema	NA
Gout	NA
Hypercholesterolemia	NA
Hypertension	0.09
Jaundice	NA
Kidney disease	0.51
Liver disease	0.72
Memory cognitive disorder	2.46
Osteoporosis	0.76
Psoriasis	0.25
Stroke	0.77
Thyroid problems	0.08
Vision	0.15

Table S4 Missing data in each variable

Variables	UK Biobank		NHANES	
	**Available values n (%)**	**Missing values n (%)**	**Available values n (%)**	**Missing values n (%)**
Multiple Long-term conditions	500612 (100.00%)	0 (0.00%)	10714 (100.00%)	0 (0.00%)
Periodontal diseases	500612 (100.00%)	0 (0.00%)	10714 (100.00%)	0 (0.00%)
Age	500612 (100.00%)	0 (0.00%)	10714 (100.00%)	0 (0.00%)
BMI	497589 (99.40%)	3023 (0.60%)	10649 (99.39%)	65 (0.61%)
Education	477329 (95.35%)	23283 (4.65%)	NA	NA
Household income	424359 (84.77%)	76253 (15.23%)	NA	NA
Smoking	497866 (99.45%)	2746 (0.55%)	10714 (100.00%)	0 (0.00%)
Physical activity	489961 (97.87%)	10651 (2.13%)	10714 (100.00%)	0 (0.00%)
Gender	500612 (100.00%)	0 (0.00%)	10714 (100.00%)	0 (0.00%)
Townsend deprivation index	499988 (99.88%)	624 (0.12%)	NA	NA
Ethnicity	498018 (99.48%)	2594 (0.52%)	10714 (100.00%)	0 (0.00%)
Marital status	403890 (80.68%)	96722 (19.32%)	10707 (99.93%)	7 (0.07%)
Acculturation score	NA	NA	10714 (98.63%)	149 (1.37%)
Family poverty level	NA	NA	10565 (100.00%)	0 (0.00%)
WBC	476407 (95.16%)	24205 (4.84%)	10348 (96.58%)	366 (3.42%)
Platelet	476409 (95.17%)	24203 (4.83%)	10347 (96.57%)	367 (3.43%)
Lymphocyte	475533 (94.99%)	25079 (5.01%)	10329 (96.41%)	385 (3.59%)
Monocyte	475533 (94.99%)	25079 (5.01%)	10329 (96.41%)	385 (3.59%)
Neutrophils	475533 (94.99%)	25079 (5.01%)	10329 (96.41%)	385 (3.59%)
CRP	466830 (93.25%)	33782 (6.75%)	3602 (33.62%)*	7112 (66.38%)*
ALT	467660 (93.42%)	32952 (6.58%)	10185 (95.06%)	529 (4.94%)
AST	466065 (93.10%)	34547 (6.90%)	10183 (95.04%)	531 (4.96%)

*CRP data only available in NHANES 2009-2010

CRP=C-reactive protein; ALT=alanine transaminase; AST=aspartate transaminase; WBC=white blood cells

Table S5 Association between periodontal disease and MLTCs: UKB and NHANES full models

UKB					NHANES				
**Variable/Category**	**Crude OR (95% CI)**	***p*-value**	**Adjusted OR (95% CI)**	***p*-value**	**Variable/Category**	**Crude OR (95% CI)**	***p*-value**	**Adjusted OR (95% CI)**	***p*-value**
**Periodontal condition**					**Periodontal disease**				
No condition	Ref		Ref		No periodontitis	Ref		Ref	
At least one condition	1.09 (1.08–1.11)	<0.001	1.12 (1.10–1.14)	<0.001	Periodontitis	1.49 (1.39–1.61)	<0.001	1.22 (1.09–1.37)	0.001
**Age**					**Age**				
≤60 years	Ref		Ref		≤60 years	Ref		Ref	
>60 years	2.84 (2.80–2.87)	<0.001	2.35 (2.32–2.38)	<0.001	>60 years	3.82 (3.37–4.33)	<0.001	3.57 (3.12–4.09)	<0.001
**BMI (continuous)**					**BMI (continuous)**				
	1.09 (1.09–1.09)	<0.001	1.08 (1.08–1.08)	<0.001		1.04 (1.04–1.05)	<0.001	1.05 (1.04–1.06)	<0.001
**Sex**					**Sex**				
Female	Ref		Ref		Male	Ref		Ref	
Male	1.16 (1.15–1.18)	<0.001	1.11 (1.09–1.12)	<0.001	Female	1.48 (1.36–1.61)	<0.001	1.44 (1.31–1.59)	<0.001
**Education**					**Education**				
University/college	Ref		Ref		College or above	Ref		Ref	
Less than high school	2.43 (2.39–2.47)	<0.001	1.27 (1.24–1.29)	<0.001	Less than high school	1.26 (1.09–1.45)	0.002	1.10 (0.92–1.32)	0.276
High school graduate	1.18 (1.16–1.19)	<0.001	0.98 (0.96–0.99)	0.001	High school	1.18 (1.03–1.35)	0.017	1.02 (0.89–1.17)	0.741
**Household income**					**Family poverty level**				
>£100,000	Ref		Ref		<100%	1.45 (1.27–1.67)	<0.001	1.35 (1.17–1.56)	<0.001
<£18,000	4.00 (3.88–4.13)	<0.001	2.38 (2.30–2.46)	<0.001	100–199%	1.49 (1.25–1.77)	<0.001	1.19 (1.00–1.41)	0.046
£18,000–£30,999	2.48 (2.41–2.56)	<0.001	1.73 (1.68–1.79)	<0.001	200–400%	1.06 (0.94-1.20)	0.335	0.88 (0.78–0.99)	0.035
£31,000–£51,999	1.71 (1.66–1.76)	<0.001	1.43 (1.38–1.47)	<0.001	>400%	Ref		Ref	
£52,000–£100,000	1.24 (1.20–1.28)	<0.001	1.17 (1.13–1.21)	<0.001	**Smoking**				
**Smoking status**					Non-smoking	Ref		Ref	
Never smoked	Ref		Ref		Smoking	1.16 (1.02–1.32)	0.030	1.27 (1.09–1.46)	0.002
Previous smoker	1.55 (1.53–1.57)	<0.001	1.30 (1.29–1.32)	<0.001	**Physical activity**				
Current smoker	1.37 (1.34–1.39)	<0.001	1.27 (1.25–1.30)	<0.001	Top third	Ref		Ref	
**Physical activity**					Bottom two thirds	1.58 (1.40-1.77)	<0.001	1.25 (1.10–1.42)	0.001
High	Ref		Ref		**Marital status**				
Low	1.28 (1.26–1.29)	<0.001	1.22 (1.21–1.24)	<0.001	Married	Ref		Ref	
**Marital status**					Widowed	4.31 (3.28–5.68)	<0.001	1.88 (1.40–2.52)	<0.001
No	Ref		Ref		Divorced/Separated	1.41 (1.25–1.59)	<0.001	1.27 (1.11–1.45)	0.001
Yes	1.01 (0.99–1.03)	0.319	1.06 (1.04–1.08)	<0.001	Never married	1.05 (0.89–1.25)	0.547	1.17 (0.99–1.39)	0.070
**Townsend deprivation**					Living with partner	0.85 (0.66–1.10)	0.206	0.92 (0.72–1.19)	0.529
Least deprived	Ref		Ref		**Acculturation score**				

Second least deprived	1.06 (1.04–1.08)	<0.001	1.00 (0.99–1.02)	0.667	US born	Ref		Ref	
Middle	1.10 (1.08–1.12)	<0.001	1.00 (0.98–1.02)	0.969	Foreign born, <10 yrs US	1.22 (0.94–1.60)	0.135	1.24 (0.94–1.64)	0.119
Second most deprived	1.14 (1.12–1.16)	<0.001	1.01 (0.99–1.03)	0.325	Foreign born, 10–19 yrs	2.11 (1.69–2.63)	<0.001	1.71 (1.34–2.19)	<0.001
Most deprived	1.41 (1.38–1.43)	<0.001	1.12 (1.09–1.14)	<0.001	Foreign born, ≥20 yrs	2.22 (1.85–2.67)	<0.001	1.66 (1.31–2.09)	<0.001
**Ethnicity**					**Ethnicity**				
White	Ref		Ref		Non-Hispanic White	Ref		Ref	
Mixed	0.80 (0.75–0.86)	<0.001	0.92 (0.85–0.99)	0.029	Mexican American	0.65 (0.54–0.78)	<0.001	0.66 (0.53–0.82)	<0.001
Asian/Asian British	0.99 (0.95–1.03)	0.606	1.07 (1.03–1.12)	0.002	Other Hispanic	0.86 (0.72–1.03)	0.090	0.96 (0.76–1.21)	0.712
Black/Black British	0.85 (0.81–0.89)	<0.001	0.76 (0.72–0.79)	<0.001	Non-Hispanic Black	0.96 (0.84–1.10)	0.570	0.79 (0.69–0.90)	0.001
Other	0.70 (0.66–0.74)	<0.001	0.78 (0.73–0.82)	<0.001	Other Race	0.76 (0.66–0.88)	0.001	1.01 (0.82–1.25)	0.902

OR = Odds Ratio, CI = Confidence Interval

Table S6 Association between periodontal diseases and MLTCs

Exposures	MLTCs Outcomes															
	MLTCs		Inflammatory-related MLTCs		Number of chronic conditions						MLTCs severity					
					2		3		≥4		Q2		Q3		Q4	
	Crude OR	Adjusted OR	Crude OR	Adjusted OR	Crude RRR	Adjusted RRR	Crude RRR	Adjusted RRR	Crude RRR	Adjusted RRR	Crude RRR	Adjusted RRR	Crude RRR	Adjusted RRR	Crude RRR	Adjusted RRR
UK Biobank																
Any periodontal conditions	1.094 (1.078, 1.110)	1.118 (1.101, 1.136)	1.086 (1.071–1.102)	1.112 (1.095–1.130)	1.040 (1.021–1.060)	1.061 (1.040, 1.081)	1.076 (1.052–1.100)	1.113 (1.088, 1.139)	1.176 (1.153–1.200)	1.228 (1.201, 1.255)	1.077 (1.055-1.100)	1.066 (1.043-1.088)	1.137 (1.113-1.160)	1.121 (1.097-1.145)	1.112 (1.09-1.136)	1.166 (1.140-1.192)
Bleeding gum	0.959 (0.943–0.975)	1.063 (1.044–1.081)	0.956 (0.941–0.972)	1.062 (1.043–1.081)	0.967 (0.947–0.988)	1.028 (1.005–1.051)	0.969 (0.945–0.993)	1.079 (1.051–1.108)	0.941 (0.919–0.963)	1.117 (1.088–1.145)	1.052 (1.029–1.077)	1.072 (1.047–1.097)	1.028 (1.004–1.051)	1.079 (1.053–1.104)	0.909 (0.888–0.930)	1.068 (1.042–1.095)
Painful gum	1.669 (1.614–1.726)	1.607 (1.551–1.666)	1.615 (1.562–1.669)	1.554 (1.500–1.610)	1.294 (1.237–1.353)	1.294 (1.237–1.354)	1.544 (1.470–1.621)	1.539 (1.463–1.619)	2.252 (2.162–2.346)	2.205 (2.108–2.306)	1.214 (1.153–1.279)	1.177 (1.117–1.240)	1.609 (1.532–1.690)	1.502 (1.428–1.579)	1.965 (1.874–2.059)	1.885 (1.793–1.982)
Loose teeth	1.416 (1.377–1.455)	1.122 (1.089–1.156)	1.416 (1.378–1.456)	1.124 (1.092–1.158)	1.225 (1.181–1.270)	1.073 (1.033–1.114)	1.361 (1.306–1.418)	1.101 (1.055–1.149)	1.704 (1.644–1.766)	1.210 (1.164–1.258)	1.136 (1.090–1.185)	1.000 (0.958–1.044)	1.407 (1.352–1.465)	1.122 (1.076–1.170)	1.676 (1.612–1.743)	1.232 (1.182–1.284)
NHANES																
Periodontitis	1.493 (1.387–1.606)	1.220 (1.089–1.367)	1.422 (1.325–1.526)	1.171 (1.048–1.309)	1.249 (1.107–1.408)	1.114 (0.965–1.286)	1.683 (1.459–1.941)	1.413 (1.164–1.714)	1.819 (1.622–2.040)	1.304 (1.132–1.502)	1.209 (1.046–1.397)	1.150 (0.977–1.353)	1.584 (1.381–1.818)	1.295 (1.119–1.499)	1.761 (1.577–1.965)	1.386 (1.247–1.541)
Periodontitis severity																
Mild	1.047 (0.829–1.324)	1.082 (0.810–1.445)	0.996 (0.801–1.239)	1.038 (0.789–1.367)	1.008 (0.742–1.369)	1.022 (0.732–1.427)	1.359 (0.958–1.927)	1.421 (0.929–2.174)	0.816 (0.537–1.239)	0.835 (0.574–1.213)	1.020 (0.648–1.607)	1.075 (0.664–1.739)	1.198 (0.888–1.616)	1.160 (0.852–1.580)	0.983 (0.709–1.362)	1.072 (0.749–1.535)
Moderate	1.629 (1.496–1.774)	1.275 (1.134–1.433)	1.585 (1.450–1.732)	1.245 (1.102–1.407)	1.299 (1.136–1.484)	1.136 (0.981–1.315)	1.862 (1.590–2.181)	1.485 (1.218–1.810)	2.095 (1.858–2.363)	1.412 (1.217–1.639)	1.211 (1.020–1.437)	1.135 (0.945–1.363)	1.659 (1.449–1.900)	1.334 (1.153–1.543)	2.013 (1.761–2.300)	1.481 (1.304–1.683)
Severe	1.303 (1.117–1.519)	1.101 (0.896–1.354)	1.142 (0.991–1.317)	0.974 (0.801–1.184)	1.226 (1.014–1.482)	1.102 (0.865–1.404)	1.274 (0.987–1.644)	1.104 (0.819–1.489)	1.496 (1.191–1.878)	1.155 (0.849–1.573)	1.324 (1.011–1.734)	1.294 (0.952–1.758)	1.574 (1.256–1.973)	1.247 (0.960–1.620)	1.387 (1.151–1.671)	1.194 (0.942–1.512)
CAL																
% sites CAL ≥3 mm	1.010 (1.008–1.011)	1.005 (1.002–1.007)	1.009 (1.007–1.010)	1.004 (1.002–1.006)	1.006 (1.003–1.008)	1.003 (0.999–1.006)	1.011 (1.009–1.014)	1.008 (1.005–1.011)	1.014 (1.012–1.016)	1.007 (1.003–1.011)	1.005 (1.002–1.008)	1.003 (1.000–1.007)	1.010 (1.008–1.013)	1.006 (1.003–1.008)	1.013 (1.011–1.016)	1.008 (1.006–1.011)

% sites CAL ≥4 mm	1.011 (1.008–1.013)	1.005 (1.002–1.008)	1.010 (1.007–1.012)	1.004 (1.001–1.007)	1.006 (1.003–1.010)	1.003 (0.999–1.007)	1.013 (1.010–1.016)	1.008 (1.004–1.012)	1.015 (1.012–1.018)	1.006 (1.001–1.011)	1.007 (1.002–1.011)	1.004 (1.000–1.009)	1.013 (1.010–1.016)	1.007 (1.004–1.010)	1.015 (1.012–1.018)	1.009 (1.006–1.012)
% sites CAL ≥5 mm	1.011 (1.007–1.016)	1.005 (1.000–1.009)	1.010 (1.006–1.013)	1.003 (0.999–1.008)	1.008 (1.002–1.013)	1.003 (0.998–1.009)	1.014 (1.009–1.019)	1.008 (1.002–1.013)	1.015 (1.010–1.020)	1.005 (0.998–1.011)	1.008 (1.002–1.014)	1.005 (0.999–1.011)	1.014 (1.009–1.019)	1.007 (1.003–1.011)	1.016 (1.012–1.020)	1.008 (1.004–1.013)
% sites CAL ≥6 mm	1.012 (1.005–1.018)	1.004 (0.998–1.011)	1.009 (1.004–1.015)	1.003 (0.997–1.009)	1.008 (1.001–1.016)	1.004 (0.996–1.011)	1.014 (1.006–1.021)	1.007 (1.000–1.015)	1.015 (1.008–1.021)	1.003 (0.996–1.010)	1.010 (1.001–1.019)	1.006 (0.998–1.015)	1.016 (1.008–1.023)	1.008 (1.002–1.014)	1.015 (1.009–1.022)	1.007 (1.001–1.013)
PPD																
% sites PPD ≥4 mm	1.005 (1.000–1.010)	1.004 (0.997–1.011)	1.001 (0.996–1.006)	1.000 (0.994–1.007)	1.007 (1.001–1.012)	1.006 (0.999–1.013)	1.004 (0.996–1.013)	1.003 (0.992–1.013)	1.004 (0.997–1.011)	1.001 (0.991–1.011)	1.005 (0.997–1.013)	1.004 (0.995–1.013)	1.016 (1.009–1.023)	1.008 (1.001–1.016)	1.002 (0.996–1.008)	1.002 (0.995–1.009)
% sites PPD ≥5 mm	1.003 (0.992–1.013)	1.001 (0.988–1.014)	0.996 (0.986–1.006)	0.995 (0.983–1.008)	1.005 (0.994–1.017)	1.004 (0.991–1.017)	1.000 (0.984–1.017)	0.999 (0.980–1.018)	1.000 (0.988–1.012)	0.998 (0.980–1.015)	1.002 (0.988–1.017)	1.001 (0.985–1.018)	1.020 (1.009–1.032)	1.011 (0.998–1.024)	0.995 (0.982–1.007)	0.996 (0.982–1.010)
% sites PPD ≥6 mm	1.004 (0.985–1.023)	1.003 (0.982–1.025)	0.992 (0.974–1.010)	0.993 (0.972–1.015)	1.008 (0.988–1.029)	1.007 (0.984–1.030)	1.002 (0.976–1.029)	1.003 (0.974–1.034)	0.996 (0.976–1.017)	0.996 (0.972–1.021)	1.007 (0.981–1.034)	1.008 (0.980–1.036)	1.035 (1.014–1.057)	1.024 (1.002–1.047)	0.995 (0.972–1.019)	0.999 (0.977–1.023)

OR = Odds Ratio; RRR = Relative Risk Ratio; CAL = Clinical Attachment Level; PPD: Probing Pocket Depth

Table S7 Mediation analyses for the association between periodontal diseases and MLTCs/ number of chronic conditions

Mediators	UK Biobank				NHANES			
	Multiple Long-term conditions		Number of chronic conditions		Multiple Long-term conditions		Number of chronic conditions	
	Crude OR	Adjusted OR	Crude OR	Adjusted OR	Crude OR	Adjusted OR	Crude OR	Adjusted OR
**White Blood Cell**								
Controlled Direct Effect (CDE)	1.072 (1.057–1.087)	1.140 (1.124–1.156)	1.054 (1.047–1.061)	1.082 (1.076–1.089)	1.509 (1.396–1.631)	1.263 (1.154–1.383)	1.216 (1.178–1.256)	1.114 (1.079–1.150)
Natural Direct Effect (NDE)	1.064 (1.020–1.108)	1.136 (1.114–1.158)	1.051 (1.039–1.063)	1.081 (1.074–1.088)	1.507 (1.393–1.631)	1.263 (1.154–1.383)	1.217 (1.179–1.256)	1.114 (1.079–1.150)
Natural Indirect Effect (NIE)	1.023 (1.021–1.025)	1.011 (1.009–1.012)	1.013 (1.012–1.014)	1.006 (1.005–1.007)	1.007 (0.999–1.016)	1.002 (0.994–1.009)	1.009 (1.005–1.013)	1.005 (1.002–1.008)
Marginal Total Effect (MTE)	1.089 (1.045–1.132)	1.148 (1.126–1.171)	1.064 (1.052–1.076)	1.087 (1.080–1.094)	1.518 (1.403–1.643)	1.265 (1.156–1.385)	1.227 (1.189–1.267)	1.120 (1.085–1.156)
Proportion Mediated (%)	26.857	7.719	20.623	6.898	1.726	0.670	4.220	4.727
**Platelet**								
Controlled Direct Effect (CDE)	1.093 (1.078–1.107)	1.150 (1.134–1.165)	1.064 (1.057–1.071)	1.086 (1.080–1.093)	1.521 (1.407–1.644)	1.265 (1.156–1.385)	1.229 (1.190–1.268)	1.120 (1.085–1.156)
Natural Direct Effect (NDE)	1.093 (1.078–1.107)	1.150 (1.134–1.165)	1.064 (1.057–1.071)	1.086 (1.080–1.093)	1.512 (1.398–1.635)	1.265 (1.156–1.385)	1.227 (1.188–1.266)	1.120 (1.085–1.156)
Natural Indirect Effect (NIE)	1.001 (1.000–1.001)	1.000 (1.000–1.001)	1.000 (1.000–1.001)	1.000 (1.000–1.000)	1.004 (0.998–1.010)	1.000 (0.999–1.001)	1.000 (0.998–1.003)	1.000 (1.000–1.000)
Marginal Total Effect (MTE)	1.094 (1.079–1.108)	1.150 (1.135–1.166)	1.064 (1.058–1.071)	1.087 (1.081–1.093)	1.518 (1.404–1.642)	1.265 (1.156–1.385)	1.227 (1.189–1.267)	1.120 (1.085–1.156)
Proportion Mediated (%)	0.721	0.279	0.425	0.254	1.004	0.000	0.201	0.000
**Lymphocyte**								
Controlled Direct Effect (CDE)	1.089 (1.074–1.103)	1.151 (1.135–1.166)	1.062 (1.055–1.069)	1.087 (1.081–1.093)	1.518 (1.404–1.641)	1.263 (1.154–1.382)	1.227 (1.188–1.266)	1.120 (1.085–1.156)
Natural Direct Effect (NDE)	1.090 (1.074–1.105)	1.150 (1.135–1.166)	1.062 (1.055–1.069)	1.087 (1.080–1.093)	1.530 (1.411–1.658)	1.267 (1.157–1.388)	1.228 (1.190–1.268)	1.120 (1.085–1.156)
Natural Indirect Effect (NIE)	1.004 (1.003–1.006)	0.999 (0.999–1.000)	1.002 (1.001–1.003)	1.000 (0.999–1.000)	0.996 (0.992–1.000)	0.998 (0.995–1.001)	0.999 (0.997–1.000)	0.999 (0.998–1.000)
Marginal Total Effect (MTE)	1.094 (1.079–1.109)	1.149 (1.134–1.165)	1.064 (1.058–1.071)	1.086 (1.080–1.092)	1.523 (1.406–1.651)	1.264 (1.154–1.385)	1.226 (1.188–1.266)	1.119 (1.084–1.155)
Proportion Mediated (%)	4.754	-0.599	3.112	-0.577	-0.995	-0.933	-0.735	-0.712
**Monocyte**								

Controlled Direct Effect (CDE)	1.098 (1.084–1.113)	1.149 (1.134–1.165)	1.068 (1.062–1.075)	1.088 (1.081–1.094)	1.510 (1.397–1.633)	1.265 (1.155–1.385)	1.220 (1.182–1.260)	1.118 (1.083–1.154)
Natural Direct Effect (NDE)	1.089 (1.057–1.121)	1.145 (1.128–1.163)	1.065 (1.054–1.075)	1.086 (1.079–1.092)	1.511 (1.397–1.635)	1.266 (1.156–1.386)	1.220 (1.182–1.260)	1.118 (1.083–1.154)
Natural Indirect Effect (NIE)	0.999 (0.998–1.000)	1.002 (1.001–1.002)	0.999 (0.999–1.000)	1.001 (1.000–1.001)	1.003 (0.994–1.012)	0.997 (0.992–1.003)	1.005 (1.001–1.009)	1.001 (0.999–1.003)
Marginal Total Effect (MTE)	1.088 (1.056–1.119)	1.147 (1.130–1.165)	1.064 (1.054–1.074)	1.087 (1.080–1.093)	1.516 (1.402–1.639)	1.262 (1.153–1.382)	1.226 (1.188–1.266)	1.119 (1.084–1.155)
Proportion Mediated (%)	-1.355	1.252	-0.962	0.951	0.728	-1.092	2.409	1.151
**Neutrophil**								
Controlled Direct Effect (CDE)	1.079 (1.065–1.094)	1.141 (1.125–1.157)	1.058 (1.051–1.065)	1.083 (1.077–1.090)	1.504 (1.391–1.625)	1.257 (1.148–1.376)	1.215 (1.177–1.255)	1.111 (1.077–1.147)
Natural Direct Effect (NDE)	1.068 (1.022–1.113)	1.135 (1.110–1.159)	1.053 (1.039–1.067)	1.081 (1.073–1.089)	1.503 (1.391–1.625)	1.257 (1.148–1.376)	1.216 (1.178–1.255)	1.112 (1.077–1.148)
Natural Indirect Effect (NIE)	1.017 (1.016–1.019)	1.010 (1.009–1.012)	1.010 (1.009–1.011)	1.006 (1.005–1.007)	1.010 (1.002–1.017)	1.005 (0.998–1.013)	1.008 (1.005–1.012)	1.006 (1.003–1.010)
Marginal Total Effect (MTE)	1.086 (1.041–1.132)	1.147 (1.122–1.171)	1.064 (1.050–1.078)	1.087 (1.080–1.095)	1.518 (1.404–1.641)	1.263 (1.154–1.383)	1.226 (1.188–1.266)	1.119 (1.084–1.155)
Proportion Mediated (%)	20.891	7.624	16.364	7.014	2.317	2.210	4.038	5.611
**C-reactive protein**								
Controlled Direct Effect (CDE)	1.058 (1.043–1.074)	1.140 (1.123–1.156)	1.044 (1.037–1.052)	1.081 (1.074–1.088)	1.607 (1.405–1.839)	1.338 (1.146–1.562)	1.236 (1.172–1.303)	1.127 (1.068–1.189)
Natural Direct Effect (NDE)	1.062 (1.046–1.078)	1.141 (1.125–1.157)	1.045 (1.039–1.052)	1.082 (1.075–1.088)	1.595 (1.391–1.830)	1.344 (1.152–1.568)	1.234 (1.172–1.299)	1.129 (1.071–1.191)
Natural Indirect Effect (NIE)	1.033 (1.030–1.036)	1.008 (1.007–1.010)	1.017 (1.016–1.019)	1.004 (1.004–1.005)	1.033 (1.013–1.054)	1.010 (0.999–1.022)	1.018 (1.008–1.028)	1.006 (1.001–1.011)
Marginal Total Effect (MTE)	1.097 (1.080–1.114)	1.150 (1.134–1.166)	1.063 (1.057–1.070)	1.086 (1.080–1.093)	1.649 (1.433–1.896)	1.357 (1.163–1.584)	1.256 (1.192–1.323)	1.136 (1.078–1.198)
Proportion Mediated (%)	35.208	5.755	27.693	5.106	6.557	3.363	7.769	4.696
**ALT**								
Controlled Direct Effect (CDE)	1.091 (1.077–1.106)	1.149 (1.134–1.165)	1.063 (1.056–1.069)	1.086 (1.080–1.092)	1.524 (1.409–1.648)	1.267 (1.156–1.388)	2.492 (1.768–3.514)	1.748 (1.272–2.400)
Natural Direct Effect (NDE)	1.091 (1.077–1.106)	1.148 (1.132–1.163)	1.063 (1.056–1.069)	1.086 (1.080–1.092)	1.526 (1.411–1.651)	1.257 (1.142–1.385)	1.227 (1.189–1.267)	1.113 (1.077–1.151)
Natural Indirect Effect (NIE)	1.003 (1.001–1.004)	1.002 (1.001–1.002)	1.001 (1.001–1.002)	1.001 (1.000–1.001)	0.998 (0.995–1.001)	0.996 (0.992–1.001)	0.999 (0.997–1.000)	0.999 (0.997–1.000)
Marginal Total Effect (MTE)	1.094 (1.079–1.109)	1.150 (1.134–1.165)	1.064 (1.057–1.071)	1.087 (1.081–1.093)	1.523 (1.408–1.647)	1.253 (1.137–1.380)	1.225 (1.187–1.265)	1.112 (1.075–1.149)

Proportion Mediated (%)	3.040	1.297	2.037	0.866	-0.005	-1.608	-0.728	-1.198
**AST**								
Controlled Direct Effect (CDE)	1.099 (1.084–1.113)	1.150 (1.134–1.165)	1.066 (1.060–1.073)	1.086 (1.080–1.093)	1.523 (1.408–1.647)	1.263 (1.153–1.384)	3.153 (1.976–5.033)	2.205 (1.442–3.369)
Natural Direct Effect (NDE)	1.098 (1.083–1.113)	1.150 (1.134–1.166)	1.066 (1.059–1.073)	1.086 (1.080–1.093)	1.508 (1.373–1.656)	1.245 (1.112–1.393)	1.225 (1.183–1.268)	1.112 (1.073–1.153)
Natural Indirect Effect (NIE)	0.996 (0.995–0.997)	1.001 (1.000–1.001)	0.998 (0.998–0.999)	1.000 (1.000–1.001)	1.001 (0.997–1.006)	1.001 (0.997–1.005)	1.000 (0.999–1.002)	1.000 (0.999–1.002)
Marginal Total Effect (MTE)	1.094 (1.079–1.109)	1.151 (1.135–1.166)	1.064 (1.057–1.071)	1.087 (1.080–1.093)	1.510 (1.375–1.659)	1.246 (1.113–1.394)	1.225 (1.184–1.269)	1.113 (1.073–1.153)
Proportion Mediated (%)	-4.290	0.362	-2.790	0.250	0.004	0.340	0.217	0.286

OR = Odds Ratio
